# Investigating Avian Influenza Infection Hotspots in Old-World Shorebirds

**DOI:** 10.1371/journal.pone.0046049

**Published:** 2012-09-28

**Authors:** Nicolas Gaidet, Ahmed B. Ould El Mamy, Julien Cappelle, Alexandre Caron, Graeme S. Cumming, Vladimir Grosbois, Patricia Gil, Saliha Hammoumi, Renata Servan de Almeida, Sasan R. Fereidouni, Giovanni Cattoli, Celia Abolnik, Josphine Mundava, Bouba Fofana, Mduduzi Ndlovu, Yelli Diawara, Renata Hurtado, Scott H. Newman, Tim Dodman, Gilles Balança

**Affiliations:** 1 CIRAD-ES, UPR AGIRS, Montpellier, France; 2 Centre National d'Elevage et de Recherches Vétérinaires, Nouakchott, Islamic Republic of Mauritania; 3 CIRAD-BIOS, UMR CIRAD/INRA CMAEE, Montpellier, France; 4 Percy FitzPatrick Institute, DST/NRF Center of Excellence, University of Cape Town, Cape Town, South Africa; 5 Friedrich-Loeffler-Institut, Insel Riems, Germany; 6 OIE/FAO Reference Laboratory for Avian Influenza and Newcastle Disease, Istituto Zooprofilattico Sper.le delle Venezie, Legnaro, Italy; 7 ARC-Onderstepoort Veterinary Institute, and Department of Production Animal Studies, Faculty of Veterinary Science, University of Pretoria, Onderstepoort, South Africa; 8 National University of Science and Technology, Bulawayo, Zimbabwe; 9 Direction Nationale des Eaux et Forêts du Mali, Bamako, Mali; 10 Parc National du Banc d'Arguin, Nouakchott, Islamic Republic of Mauritania; 11 Department of Microbiology, Institute of Biomedical Science, University of São Paulo, São Paulo, Brazil; 12 Department of Preventive Medicine and Animal Health, Faculty of Veterinary Medicine University of São Paulo, São Paulo, Brazil; 13 Food & Agriculture Organisation of the United Nations, Wildlife Health & Ecology Unit, EMPRES Animal Health, Rome, Italy; 14 Wetlands International, Wageningen, The Netherlands; University of Georgia, United States of America

## Abstract

Heterogeneity in the transmission rates of pathogens across hosts or environments may produce disease hotspots, which are defined as specific sites, times or species associations in which the infection rate is consistently elevated. Hotspots for avian influenza virus (AIV) in wild birds are largely unstudied and poorly understood. A striking feature is the existence of a unique but consistent AIV hotspot in shorebirds (Charadriiformes) associated with a single species at a specific location and time (ruddy turnstone *Arenaria interpres* at Delaware Bay, USA, in May). This unique case, though a valuable reference, limits our capacity to explore and understand the general properties of AIV hotspots in shorebirds. Unfortunately, relatively few shorebirds have been sampled outside Delaware Bay and they belong to only a few shorebird families; there also has been a lack of consistent oropharyngeal sampling as a complement to cloacal sampling. In this study we looked for AIV hotspots associated with other shorebird species and/or with some of the larger congregation sites of shorebirds in the old world. We assembled and analysed a regionally extensive dataset of AIV prevalence from 69 shorebird species sampled in 25 countries across Africa and Western Eurasia. Despite this diverse and extensive coverage we did not detect any new shorebird AIV hotspots. Neither large shorebird congregation sites nor the ruddy turnstone were consistently associated with AIV hotspots. We did, however, find a low but widespread circulation of AIV in shorebirds that contrast with the absence of AIV previously reported in shorebirds in Europe. A very high AIV antibody prevalence coupled to a low infection rate was found in both first-year and adult birds of two migratory sandpiper species, suggesting the potential existence of an AIV hotspot along their migratory flyway that is yet to be discovered.

## Introduction

Heterogeneity in the transmission rates among host species and across geographical ranges is a major determinant of the dynamic of infectious diseases [1]. Particular seasons, environments, or species associations can generate disease “hotspots” in which pathogen prevalence is consistently higher than elsewhere. These hotpots play a major role in the dynamics of infectious diseases: for instance, seasonal peaks in infection rate produce a rapid increase in the level of the population immunity, affecting the long-term maintenance of a pathogen in the host population; elevated pathogen prevalence may facilitate reassortment between heterosubtypic pathogens; and hotspots may constitute a source of pathogen spillovers to less susceptible or less exposed species, environments or geographical areas that are connected to the hotspot by host movements. Identifying the occurrence of hotspots is therefore of particular importance for the control and prevention of infectious diseases.

Low pathogenic avian influenza viruses (AIV) have been extensively studied in wild birds in recent years in response to the emergence and dispersion of highly pathogenic AIV responsible for major health and economic threat [2]. Shorebirds (Charadriiformes) are classically recognised, together with ducks, geese and swans (Anseriformes), as the major natural reservoir of AIV [3], [4]. Globally and locally, the typical prevalence of AIV infection in shorebird species sampled worldwide is low (c. 1%) [3]–[6] as compared with prevalence in ducks (c. 10% globally with seasonal peaks of 20–60%) [3], [4]. There is, however, one notable exception: a high AIV prevalence (>10%) has been consistently reported in the ruddy turnstone (*Arenaria interpres*) sampled in May during spring migration at Delaware Bay, USA [5], [7], [8]. This particular species, season and site combination represent the only known shorebird-AIV hotspot worldwide at which the infection rate is consistently higher than elsewhere in the world [5], [6], [8]. Delaware Bay is one of the world's largest congregation sites of shorebirds, supporting an estimated 1 million shorebirds stopping during spring migration. Surprisingly, all of the other shorebird species that mingle with the ruddy turnstones at Delaware Bay in May show a very low AIV prevalence (<2%) [5], [9]. A low prevalence has also been found in shorebirds (including ruddy turnstone) stopping at Delaware Bay during autumn migration [7], [10].

Shorebirds form the most abundant and the most species-rich group of waterbirds. Shorebirds are divided taxonomically into three major clades: the Scolopaci (sandpipers, snipes, phalaropes, jacanas), Lari (gulls, terns, auks, pratincoles, skuas) and Charadrii (plovers, oystercatchers, stilts) [11]. Most species share a set of ecological characteristics that are favorable to the transmission and dispersion of AIV: (i) they are generally highly gregarious (at least outside the breeding season), congregating at very high densities at key staging sites along migratory flyways, where they form multispecies foraging or roosting flocks; (ii) most species are very long-distance migrants, including some that undertake non-stop flights of up to 11,000 km [12]; and (iii) many shorebirds breeding in the Northern hemisphere winter in the Southern hemisphere; in this way they connect the northern hemisphere waterbird fauna to the regions south of the equator that are not reached by migratory ducks.

Characterisation of AIVs isolated from shorebirds suggests that they play a key role in the perpetuation of AIV in wild bird communities. The majority of inter-continental exchange and reassortment of AIV genes between Nearctic and Palaearctic regions has, although uncommon, been reported in shorebirds [13], [14]. A larger variety of hemagglutinin and neuraminidase combination subtypes has been found in shorebirds than in ducks [8], [10] suggesting that shorebirds may maintain a wider spectrum of viruses than ducks [3]. Furthermore, phylogenetic studies consistently indicate the existence of a large overlap between the gene pools of viruses that originate from both ducks and shorebirds [13], [15], suggesting that AIVs are commonly transmitted between those two groups of species.

It is not currently known whether Delaware Bay constitutes the world's only shorebird AIV hotspot or whether other hotspots exist, in particular at other large shorebird congregation sites [5], [6]. Published studies have limitations in their geographic scope, numbers of sampled birds, taxonomic range, and ability to detect AIVs. So far there have been relatively few intensive studies of AIV infection in shorebirds outside Delaware Bay (see [6] for a review) and it is unclear whether the number of birds sampled at other locations and times has been adequate to detect hotspots. Most of the shorebirds that have been tested belong to only two (Scolopacidae and Laridae) of the 19 families of Charadriiformes. In addition, almost all AIV infection studies in shorebirds have relied only on cloacal or fresh fecal samples [6], [8], [16], [17], whilst oropharyngeal samples have been tested on very few occasions [8], [18]. Several recent studies of Anseriformes provide evidence of the importance of the respiratory tract for the replication of AIV [2], [19] and demonstrate the complementary nature of cloacal and oropharyngeal samples, since birds are rarely found concurrently infected from both types of samples [8], [20]. Results from experimental infection studies also suggest that oropharyngeal excretion may be predominant for some AIV subtypes in gulls [21], [22]. As a result of these various limitations, some hotspots of AIV infection in shorebirds may have remained undetected.

As this summary implies, the significance of interspecific variation in AIV prevalence among shorebirds, and in particular the high prevalence in the ruddy turnstone, remains unclear. Ruddy turnstones have rarely been tested for AIV infection outside Delaware Bay (only 9%, see supporting information Table S1). It remains to be determined whether this species is associated with AIV hotspots in other sites or if other species are involved.

**Figure 1:**
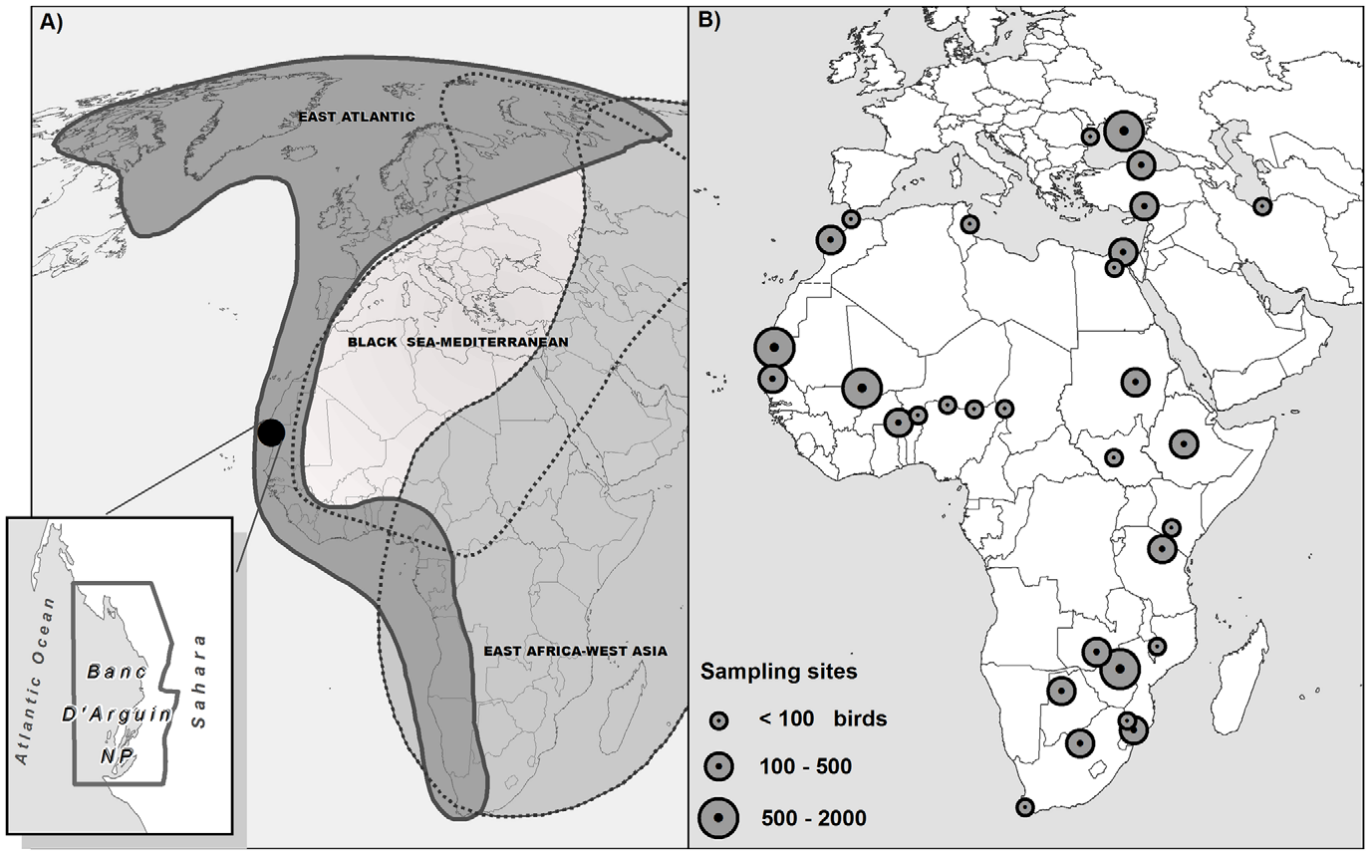
Location of the study sites. (A) The Banc d'Arguin (Mauritania) and the main shorebirds migratory flyways across Western Eurasia and Africa. (B) All shorebird sampling sites considered in our study (list of sites ranked by latitude: Ukraine - Eastern Sivash, Romania-Danube Delta, Turkey - Kizilirmak Delta and Yumurtalik Lagoons, Iran - Fereydoon Kenar marshes, Morocco - Marais du Bas Loukkos and Sidi Moussa-Oualidia Lagoon, Tunisia - Thyna salt pans, Egypt - Nile River Delta and Lake Qarun, Mauritania - Banc d'Arguin National Park, Senegal/Mauritania - Senegal River Delta, Republic of Sudan - El Saggay Island, Mali - Inner Niger Delta, Niger - Kurfunkura pond and Gaya, Chad - Lake Chad, Nigeria - Hadejia-Nguru wetlands, Burkina Faso - Lake Kompienga, Ethiopia - Lake Debre Zeit, South Sudan - Bargel wetland, Kenya - Lakes around Nairobi, Tanzania - Lake Manyara, Malawi - Lake Chilwa, Zambia - Kafue Flats, Zimbabwe - Lakes Manyame-Chivero, Botswana - Lake Ngami, Mozambique - Massingir Dam and Lake Chuali, South Africa - Barberspan wetland and Strandfontein). A detailed list of sampling sites is provided in the supporting information Table S2 (map by M. Gély ©Cirad).

In this study we investigated the possibility that other AIV hotspots may be associated with large shorebird congregation sites or with alternative species. First, we conducted a survey of AIV infection in shorebirds, including ruddy turnstone, at the Banc d'Arguin in Mauritania (Table 1, Figure 1). This site constitutes one of the largest wintering sites for shorebirds in the world (c. 2.3 million birds) and supports the greatest number of ruddy turnstones (c. 9,000 birds) across the old world [23]. Second, we extended our analysis to a large-scale dataset of AIV prevalence in 69 shorebird species that we sampled in 25 countries at some of the most important shorebirds sites in Africa and Western Eurasia (Figure 1, and in supporting information Table S2) during various international surveillance programs [20], [24], [25]. We explored these data for the presence of potential hotspots of AIV infection in relation to the number of birds sampled at a given time and location. We also examined whether variations in prevalence could be linked to ecological factors that affect (i) the local persistence and transmission potential of AIV (such as the environment and local abundance of shorebirds) and (ii) the exposure of host species to AIV (such as their geographical range and foraging behaviour). Third, we conducted a serologic survey in some shorebird species to compare differences in previous AIV exposure between species throughout their annual range and identify the species that might potentially be involved in AIV transmission at other hotspots.

**Table 1:** Prevalence of AIV infection detected by rRT-PCR in shorebirds sampled at the Banc d'Arguin, Mauritania.

Species	No. bird positive/total						% pos. (±95% CI)
	Feb 2006	Dec 2006	Apr 2008	Nov 2009	Mar 2010	Total	
Ruddy turnstone *Arenaria interpres*		1/28	0/98	0/5	0/27	1/158	0.6 (0.1–3.5)
Sanderling *Calidris alba*		0/19		0/6	0/22	0/47	0 (0–7.6)
Dunlin *C. alpina*		3/186	0/121	0/269	1/160	4/736	0.5 (0.2–1.4)
Red knot *C. canutus*		3/128		0/88	3/131	6/347	1.7 (0.8–3.7)
Lesser black-backed gull *Larus fuscus*	1/129		0/1	0/63	0/30	1/223	0.4 (0–2.4)
Slender-billed gull *Chroicocephalus genei*			1/141	0/26	0/99	1/266	0.4 (0–2.1)
Terns^a^	2/150					2/150	1.3 (0.4–4.7)
other species^b^		1/35	0/4	0/41	0/11	1/92	1.1 (0.1–5.9)
Total	3/279	8/396	1/365	0/498	4/480	16/2018	0.8 (0.5–1.3)

a . Caspian tern *Sterna caspia, Royal tern S. maxima, Sandwich tern S. sandvicensis*.

b . Curlew sandpiper *Calidris ferruginea,* Little stint *C. minuta,* Common ringed plover *Charadrius hiaticula,* Eurasian oystercatcher *Haematopus ostralegus,* Bar-tailed godwit *Limosa lapponica,* Grey plover *Pluvialis squatarola,* Common redshank *Tringa totanus,* Grey-headed gull *Chroicocephalus cirrocephalus,* Black-headed gull *C. ridibundus*.

## Methods

### Ethics statement

Birds used in this study were sampled from Botswana, Burkina Faso, Chad, Egypt, Ethiopia, Iran, Kenya, Mali, Mauritania, Morocco, Niger, Nigeria, Malawi, Mozambique, Ukraine, Senegal, Republic of Sudan, South Sudan, Tanzania, Tunisia, Turkey, Zambia and Zimbabwe (Figure 1). Most birds were captured and released in the wild using conventional techniques (mist-nets and baited walk-in traps) covered in the Ornithological Council “Guidelines to the Use of Wild Birds in Research”. Procedures for capture, handling, and sampling were approved by the Centre de Recherches par le Baguage des Populations d'Oiseaux (CRBPO, Natural History Museum Paris - French National Reference Bird Ringing Center). We conducted a serological survey on a subset of birds captured at two sampling sites (Banc d'Arguin, Mauritania; Inner Niger River Delta, Mali) (see below for details). A capture and sampling permit at the Banc d'Arguin was obtained from the Conseil Scientifique du Banc d'Arguin. Capture permits were similarly obtained from the relevant government authority in each country where field studies were conducted. All sampling activities were conducted in the presence of a representative from the animal health and veterinary national services and a representative from the environment national services.

On some occasions birds (8% of the total sample) were provided by hunters. Samples were collected from traditional or commercial safari hunting activities. Birds that had been killed by hunters had been hunted with appropriate permits from each relevant local authority and were obtained with the hunters' permission. None of the collected birds were protected. Safari operators conduct their activities through authorisations delivered by: Morocco: Haut Commissariat aux Eaux et Forêts et à la Lutte Contre la Désertification, Direction de la Lutte contre la Désertification et de la Protection de la Nature; Burkina Faso: Direction de la Faune et des Chasses; Chad: Direction de la Conservation de la Faune et des Aires Protégées. In two countries (Mali, Malawi) birds were acquired through local bird hunters who harvest birds for subsistence and to supplement and sustain the food resources of the local population. In Mali birds were acquired from local bird hunters through an official hunting and sampling authorisation delivered by the Direction National de la Conservation de la Nature (Félix Dakouo, National Director of the DNDN). In Malawi birds were acquired through registered bird hunters who belong to bird hunting clubs that form part of the Lake Chilwa Bird Hunters Association which was formed with the aim of sustainably managing the utilisation of sedentary and migratory waterbirds at Lake Chilwa; sampling authorisation was provided by the Wildlife Conservation Department, Ministry of Agriculture and Rural Development. On two sampling occasions (Niger and Burkina Faso in 2006) birds were shot through special permits for sample collection in the framework of a national emergency surveillance operation implemented after the notifications of avian influenza highly pathogenic H5N1 outbreaks. These birds (n = 163) represent only 2% of the entire birds sampled in this study. Hunting authorisations were obtained from the Direction de la Conservation de la Nature for Niger (Ali Harouna, Director of the Direction de la Conservation de la Nature) and the Direction de la Faune et des Chasses for Burkina Faso (Urbain Belemsobgo, Director of the Direction de la Faune et des Chasses).

### The Banc d'Arguin

The Banc d'Arguin National Park is located near the 20^th^ parallel and extends >180 km along the Atlantic coast of Mauritania, bordered by the Sahara desert (Figure 1). This area of about 500 km^2^ of very shallow intertidal flats is flooded by upwelling of cold water rich in nutrients. This exceptional ecosystem is the wintering site with the highest density of shorebirds along the East Atlantic Flyway [26]. It also represents a crossroads for migratory shorebirds breeding in the Palearctic, Nearctic and Afro-tropical regions and wintering along the African coast.

### Sampling and AIV detection procedures

Shorebirds were sampled at the Banc d'Arguin on five occasions between 2006 and 2010 (Table 1). Samples were collected during the same period at 30 other sites in 25 countries (Figure 1, and supporting information Table S2). Sampling was conducted on a different number of occasions according to sites and years. Three types of samples were collected that we distinguished in our subsequent analyses: a single cloacal or fecal swab, a single oropharyngeal swab, or both cloacal and oropharyngeal swabs tested separately. All samples were collected using cotton swabs and stored in cryovials containing a viral transport medium. Cryovials were stored in liquid nitrogen directly in the field and every effort was made to maintain the cold chain using cryopacks with dry ice during international shipment to the laboratory. Samples were analysed in different laboratories using a similar standard diagnostic procedure based on RNA extraction and real-time RT-PCR virus detection (rRT-PCR) targeting the matrix gene specific for influenza A viruses. Positive samples were also inoculated into embryonated SPF chicken eggs for virus isolation and typed according to standard procedures. Further details on sampling and diagnostic procedures can be found in a previous publication [20]. We computed the observed prevalence for each species for each sampling occasion as the percentage of individuals found positive for AIV compared with the total number of birds tested.

### Presence of hotspots of AIV infection

We defined a hotspot as a specific location and time at which the AIV infection rate is consistently elevated and about an order of magnitude greater than in other sites, using 10% as a minimum prevalence threshold according to long-term prevalence measures in ruddy turnstone at Delaware Bay [5], [8]. We compared the number of AIV-positive birds detected on each sampling occasion (considering birds from all species altogether or from each species separately) with a threshold number of positive birds for a sample of the same size below which the prevalence is unlikely (probability <0.05) to be greater than 10%. This threshold was defined as the 0.05 quantile of a binomial distribution B(n = sample size, p = 0.10) (*stats* R package, *qbinom* procedure). If the observed number of AIV-positive birds on a sampling occasion was below the threshold defined for a sample of the same size, we concluded that prevalence in the population from which the sample had been drawn was most likely lower than 10%. We restricted our analysis to sampling units that had at least 28 birds sampled (28 being the minimum number of individuals required to be 95% confident of detecting at least one infected bird when prevalence is ≥10%).

### Variations in AIV prevalence in shorebirds across Eurasian and Afro-tropical regions

Explanatory variables tested in this analysis are summarized in Table 2. Species behavioral and ecological traits were taken from literature (body mass, main foraging technique: [27], [28]; geographic range: [23]). We restricted our analysis to species that had at least 20 individuals sampled. Sampling sites were classified according to four abundance classes of shorebird populations estimated from counts compiled in [23].

**Table 2:** List of the explanatory variables tested to explain geographical, seasonal and species variations in AIV prevalence in shorebirds across Eurasian and Afro-tropical regions (Figure 1).

Explanatory variables		Eco-epidemiological predictions	Definition
Site	Shorebirds abundance	Aggregation of birds may enhance inter-individual transmission through contact rate	Four abundance classes ([<5], [5–50], [50–500], [>500]×10^3^ birds)
	Environment	High salinity, wind and solar radiation exposure (low vegetation cover) and tidal washing may reduce virus persistence in coastal habitat	Marine-saline vs inland-freshwater habitats
Season		Seasonal patterns of migration and reproduction may influence the turnover of susceptible birds	Four trimester periods
Sampling procedure		AIV may replicated preferentially in the respiratory or the digestive tract; the type of sample tested may influence the probability of detecting an infection	Single cloacal, single oropharyngeal or both types of samples
Species	Foraging behaviour^a^	Higher AIV exposure in tactile-foraging (probing) than in visual-foraging (pecking) species	Tactile-foraging vs visual-foraging species
	Geographic range^a^	Lower AIV exposure in high arctic/coastal than boreal-temperate/freshwater species, and in Afro-tropical resident than in boreal-temperate migratory species	High arctic/coastal vs boreal-temperate/freshwater vs tropical/freshwater species
	Body mass^a^	Demographic rates associated with body mass may influence the turnover of susceptible birds	Mean species body mass

a . Only tested for species from the Scolopaci and Charadrii clades.

We explored the relationships between AIV prevalence and explanatory variables using Generalized Linear Mixed Models (GLMM) with a binomial error distribution and a logit link function (*lme4* R package, *glmer* procedure). We tested the independence among categorical variables (phi coefficient) to identify potential collinearity issues. Samples had usually been collected on several occasions at the same site or during the same year; to avoid pseudo-replication we included a year and a site random effect in models. The potential aggregation of infected birds within a given sampling occasion was also accounted for by incorporating the sampling occasion as a random effect nested within year and site. Finally, we included a random laboratory effect to account for a potential difference in diagnostic sensitivity among laboratories.

Our analysis consisted of two steps. First, we tested for environmental, seasonal and species variation in prevalence, accounting for differences in the types of sample tested. The two variables related to the sampling site (abundance and environment) were considered associated (phi coefficient >0.28) and were tested alternatively in models. We constructed a full model including four explanatory variables (species, season, sampling procedure and abundance/or environment,) as fixed effects and four random factors (year, site, sampling occasion and laboratory). The goodness-of-fit of the model was assessed using the Pearson Chi-Square statistic. The initial full model was simplified by a stepwise backwards elimination of non-significant variables. We used likelihood ratio tests (LRT) to test the significance of each variable, computing the χ2 of the LRT between the model retaining and the model excluding the variable. We first explore the random part of the model, iteratively removing from the model the random factors with the lower estimated variance components. We then explored the fixed part, iteratively removing variables that had the lowest explanatory power (highest P values, t-test) to identify the minimal adequate model.

In a second step we tested whether species variation in prevalence was related to species traits that may affect exposure and immunity to re-infection (Table 2). In most ecosystems, cohabiting shorebird species are separated in space and time through dietary preferences and foraging techniques leading to potential differences in exposure to infection. Shorebirds of the Scolpaci (sandpipers and allies) and Charadrii (plovers and allies) clades use two main sensory mechanisms to detect their prey: species relying on tactile sensation that forage mainly by probing prey continuously beneath the substrates may have a higher AIV exposure by comparison to species that use visual mechanisms and forage by pecking prey from the surface. Moreover, differences in foraging strategy lead to variations in vigilance; tactile-foraging species tend to form larger foraging flocks than visual-foraging species [29] and hence may be subject to higher contact rates and inter-individual AIV transmission. Species heterogeneity in transmission rate may be caused by latitudinal and habitat differences in AIV exposure. Environmental persistence of AIV is reduced by salinity. A lower AIV exposure is expected in the high Arctic regions where shorebirds breed at low density, forage mainly on terrestrial invertebrates in moist or dry habitat and where the dabbling ducks of the *Anas* genus (presumably the main AIV maintenance hosts [3]) are largely absent. High Arctic breeding shorebird species winter predominantly in coastal-saline environments [30] and therefore remain year-round in AIV-poor environments, whereas species breeding in sub-Arctic to temperate regions winter mainly in inland-freshwater wetlands where the potential for AIV infection is higher (freshwater, cohabitation with dabbling ducks of the *Anas* genus). Eurasian migratory species wintering in Africa move between vastly separated areas with distinct waterbird communities and hence are likely to be exposed to a higher diversity of AIVs than Afro-tropical species that reside year-round within sub-Saharan regions where AIVs generally circulate at a lower level than in temperate or boreal wetlands [20].

In the second step we followed the same model selection procedure as in the first step, starting from the previous minimum adequate model but substituting the variable “species” with variables describing species traits. Since demographic turnover rate in birds is related to body size at the inter-specific level, we included among the potential explanatory variables body mass (log transformed) as a proxy for turnover rate of susceptible birds in the population. In order to account for non-independence between species owing to phylogeny we included a hierarchical taxonomic (Clade–Family–Genus–Species) nested error structure as random effects in the model. The foraging and range classes defined for the Scolopaci and Charadrii were not appropriate for species belonging to the Lari clade (most gull and tern species that we sampled remained year-round in coastal habitats and forage using either specialized techniques, such as aerial-plunging in terns, or opportunism in gulls); they were removed from this second analysis.

### Serological survey of antibodies to AIV

We conducted a serological survey on a subset of birds captured at two sampling sites (Banc d'Arguin, Mauritania; Inner Niger Delta, Mali) during three sampling occasions at each site. Birds were ringed and aged (first year, ≥second year, undetermined) on the basis of plumage characteristics, and a blood sample was collected from the jugular vein. We tested individual serum samples for the presence of antibodies specific to the AIV nucleoprotein by using a commercial blocking enzyme-linked immunosorbent assay (bELISA) kit (FlockCheck AI MultiS-Screen nucleoprotein antibody test kit, IDEXX Laboratories) following the manufacturer's instructions. All samples were run in combination with supplied positive and negative controls for validation. Optical density (OD) values were read at 650 nm and sample with S/N values (ratio of the sample OD to the kit negative control mean OD)<0.50 were considered positive for antibodies to AIV.

We also tested a subset of samples from different species at both sites using a second commercially competitive ELISA kit (ID screen, influenza A nucleoprotein antibody competition, ID.VET) following the manufacturer's instructions. We evaluated the reproducibility of diagnostic results using i) the McNemar's test (exact binomial test for correlated proportions) to assess if proportions of positive results differed between the two Elisa assays, and ii) the Cohen's Kappa statistic to measure the level of agreement between the two tests beyond chance. Seroprevalence of antibodies to AIV was computed for each species and sampling occasion from results of the first diagnostic assay.

We investigated the variations in seroprevalence using a GLMM approach, as described above. We tested for effects of age and species as fixed factors, accounting for potential pseudo-replication issues by including year, site and sampling occasions as random factors. We also restricted this analysis to species that had at least 20 individuals sampled.

## Results

### AIV circulation at PNBA, Mauritania

A total of 2018 shorebirds were sampled at the Banc d'Arguin. AIV was detected on four out of the five sampling occasions and in almost all species, but at a consistently low prevalence (<2%, Table 1). Only one AIV-positive ruddy turnstone was found, and the proportion of birds found infected with AIV in this species (0.6%, n = 158) was not significantly different from that in other shorebird species (0.8%, n = 1860) (χ2 = 0.06, *p*>0.5). We also found no difference in infection rate for all shorebird species between sampling occasions conducted at the beginning (Nov–Dec: 0.9%, n = 894) and the end (Feb–Apr: 0.7%, n = 1124) of the wintering period (χ2 = 0.21, *p*>0.5).

### Presence of AIV hotspots in shorebirds across Eurasian and Afro-tropical regions

Altogether, a total of 7715 birds belonging to 69 species from 10 shorebird families were tested for AIV infection during 86 sampling occasions at 31 sites (in supporting information Table S3). The global prevalence was low: AIV infection was detected in only 1.4% (n = 107) of birds tested. Two AIVs were isolated, an H1N1 virus in dunlin (*Calidris alpina*) in Turkey and an H10N7 virus in Spotted Redshank (*Tringa erythropus*) in Romania.

**Figure 2:**
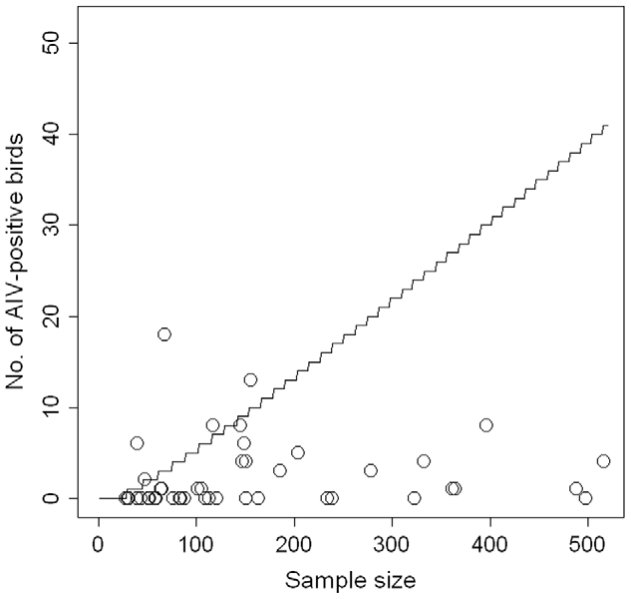
Detection of potential hotspots of AIV infection in shorebirds sampled at various sites across Eurasian and Afro-tropical regions (**Figure 1**-B; and supporting information Table S2). The number of AIV-positive birds detected in relation to the number of birds sampled per sampling occasion is here compared to the threshold number of positive birds (solid line) below which the prevalence is unlikely (probability <0.05) to be greater than 10% for a sample of the same size. Points on or above the line represent potential AIV hotspots, i.e. sampling occasions (n = 5) for which the number of positive birds was too large for rejecting the hypothesis that prevalence could be >10% (see Table 3). Only sampling occasions (n = 47) that had at least 28 birds sampled were considered in this analysis.

Our analysis for the presence of potential AIV hotspots in the shorebirds revealed that on most sampling occasions, the number of AIV-positive birds detected was below the threshold number of infected individuals expected for a prevalence of at least 10% (Figure 2). For about 90% of sampling occasions when at least 28 birds were tested (42 out of 47 occasions) we can be 95% confident that prevalence was lower than 10%. We found similar results when we considered each shorebird species separately (supporting information Figure S1).

On only five sampling occasions (Table 3) the number of positive birds was too large for rejecting the hypothesis that prevalence could be at least 10%. For these, we could not exclude the presence of a hotspot of AIV infection. Interestingly, three of these sampling occasions originated from the same site (Lakes Manyame-Chivero, Zimbabwe) and from the same bird species (mainly African jacana *Actophilornis africana* and Blacksmith lapwing *Vanellus armatus*), although from three distinct sampling seasons (Sep 2007, Jan 2008 and Nov 2008). The two other sampling occasions were in the Senegal River Delta and at the Thyna Salt Pans (Gulf of Gabès) in Tunisia. Sampling conducted on these same three sites at the same time-point and on the same species on successive years did not, however, confirm the recurrent presence of a hotspot of AIV infection at these specific locations and times of the year (Table 3).

**Table 3:** List of potential hotspots of AIV infection detected in our study.

Country	Site	Occasion	Main species^a^	No. bird pos./total	% pos. (±95% CI)
*Potential AIV hotspots*					
Zimbabwe	Lakes Manyame-Chivero	Sep. 2007	African jacana, Kittlitz's plover, Little stint	6/40	15.0 (7.1–29.1)
		Jan. 2008	African jacana, Blacksmith lapwing	18/68	26.5 (17.4–38.0)
		Nov. 2008	African jacana, Blacksmith lapwing, Kittlitz's plover	8/117	6.8 (3.5–12.9)
Mauritania-Senegal	Senegal River Delta	Mar. 2006	Slender-billed gull	13/156	8.3 (4.9–13.7)
Tunisia	Thyna salt pans	May 2006	Curlew sandpiper	2/48	4.2 (1.1–14.0)
*Follow-up sampling at the same site and month in the successive years*					
Zimbabwe	Lakes Manyame-Chivero	Sep. 2008	African jacana, Kittlitz's plover	0/77	0 (0–4.8)
		Jan. 2009	African jacana, Blacksmith lapwing	0/84	0 (0–4.4)
		Nov. 2009	African jacana, Wood sandpiper, Blacksmith lapwing	1/102	1.0 (0.0–5.3)
		Fev. 2010	African jacana	1/64	1.6 (0.1–8.3)
Mauritania-Senegal	Senegal River Delta	Mar. 2010	Slender-billed gull	0/36	0 (0–9.6)
Tunisia	Thyna salt pans	Apr. 2007	Little stint, Curlew sandpiper	0/44	0 (0–8.0)

These sites correspond to sampling occasions (top) at which the number of AIV-positive birds was above the threshold number of birds for which the hypothesis that prevalence is lower than 10% could not be rejected (see Figure 2). Sampling conducted in different years at the same sites during the same months and on the same species (below) detected a low number of AIV-positive birds.

a . African jacana *Actophilornis africana*, Kittlitz's plover *Charadrius pecuarius*, Little stint *Calidris minuta*, Blacksmith lapwing *Vanellus armatus*, Slender-billed gull *Chroicocephalus genei*, Curlew sandpiper Calidris ferruginea, Wood sandpiper *Tringa glareola*.

### Variations in AIV prevalence in shorebirds across Eurasian and Afro-tropical regions

Although AIV prevalence was generally low, AIV infection was relatively ubiquitous in shorebirds across Eurasian and Afro-tropical regions. AIV positive individuals were found in a large number of bird species (n = 26) of both Eurasian and Afro-tropical origin, belonging to various families including Scolopacidae (13 species), Charadriidae (8), Laridae (7), Jacanidae (1) and Glareolidae (1) (in supporting information Table S3). Infection with AIV was reported for the first time in about two thirds (n = 18) of these species. AIVs were also detected in birds sampled throughout the year, in all study regions, and in both inland-freshwater and coastal-saline environments.

In our first analysis of the environmental, seasonal and species variations in prevalence, we found that the sampling occasion accounted for most of the variance in the random part (random effect variance estimation = 4.47). The inclusion of each of the other random factors (laboratory, site and year) did not improve the model fit (LRT, p>0.5). We therefore included sampling occasion as a random effect in all subsequent models. During our selection procedure among explanatory variables we found that only sampling procedure and species had a significant effect on variation in prevalence (Table 4). Prevalence was significantly higher for birds tested concurrently for both cloacal and oropharyngeal samples than in birds tested for a single cloacal sample (Z value = 2.29, p = 0.022). However, prevalence was similar between birds tested for a single cloacal or a single oropharyngeal sample (Z value = 0.93, p = 0.35). We detect a significant variation in prevalence between species but no species had prevalence significantly different from the prevalence estimated for the ruddy turnstone. We did not detect a statistical difference in prevalence between seasons, the type of environment or the abundance classes of shorebirds at the sampling site (Table 4).

**Table 4:** Results of the model selection procedure relating variations in AIV prevalence in shorebirds across Eurasia and Africa to species ecological traits, sampling procedure, period and ecological characteristics of the sampling site.

Explanatory variables	Coefficient ± S.E.	χ2	df	p	
*Full model: all species*					
Sample type	Cloacal+oropharyngeal	1.59±0.70*	9.04	2	0.011
	Single oropharyngeal	0.75±0.80			
Species		NS^a^	66.1	34	<0.001
Abundance			3.06	3	0.38
Environment			0.05	1	0.82
Season			1.65	3	0.65
*Species traits: only Scolopaci and Charadrii species*					
Sample type	Cloacal+oropharyngeal	1.58±0.77*	8.03	4	0.018
	Single oropharyngeal	0.90±0.84			
Geographic range			1.90	6	0.39
Foraging behaviour			0.47	5	0.49
Body mass			0.10	5	0.75

All models were fitted as generalized mixed effects models, with sampling occasion fitted as random intercept terms to control for pseudo-replication and other explanatory variables as fixed effect. The initial full model was simplified by backwards elimination of non-significant variables. Coefficient estimates are given only for variables interpreted as statistically significant (t-test,* p<0·05) and included in the minimal adequate model (NS - no coefficient of individual species was statistically different from zero). The test statistics refer to a likelihood ratio test between the model in which the variable is retained and in which it is excluded. Statistics for variables not included in the final model correspond to the values when added to the minimal adequate model. Variables related to species traits were included in substitution to the variable species.

The variable species and sampling procedures were also significant when we restricted our analysis to Scolopaci and Charadrii. None of the variables related to species traits (geographic range, foraging behaviour and body mass), however, was a significant predictor of AIV prevalence and only the variable sampling procedure was retained in the selection process (Table 4). None of the nested taxonomic levels (Clade–Family–Genus–Species) included as a random effect received statistical support from the data and sampling occasion accounted for most of the variance of the random part (variance estimation = 4.89).

### Seroprevalence of antibodies to AIV

The presence of antibodies specific to AIV was investigated in a total of 930 birds belonging to 23 species (in supporting information Table S4). The overall seroprevalence was 17.6% and was highly variable between species, ranging from 0 to 77%. The reproducibility of diagnostic results, evaluated by testing a subset of samples (n = 258) using a second ELISA kit, was high: we found no significant difference in the proportion of positive samples between the two ELISA assays (McNemar's chi-squared = 5.79, df = 1, p-value = 0.016) with an almost perfect agreement (Kappa test = 0.87, p<0.001).

**Figure 3:**
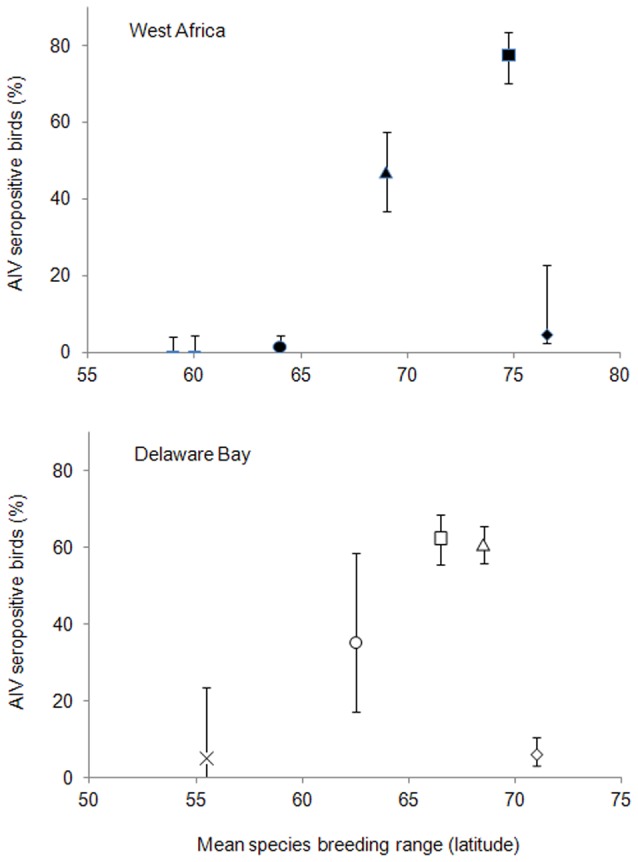
Mean seroprevalence of AIV antibodies among closely related shorebird species in relation to the mean latitude of their breeding range. Seroprevalence were measured in West Africa (the Banc d'Arguin, Mauritania and the Inner Niger Delta, Mali) and the Delaware Bay, USA (from [8], [31]) using the same commercial bELISA kit (see Methods). The mean species breeding latitude was computed from the northern and southern limits of the breeding distribution of the populations present at each site using distribution maps from [22], [32]. Species include ruddy turnstone (Δ), red knot (□), dunlin (o), sanderling (◊), short-billed dowitcher (×), ruff and wood sandpiper (−). Error bars represent the binomial exact 95% confidence interval.

Our GLMM analysis indicated that seroprevalence was unrelated to age (χ2 = 0.79, df = 12, *p* = 0.67) but varied significantly between species (χ2 = 281.31, df = 10, *p*<0.001). Age also had no significant effect when we excluded birds of unknown age (χ2 = 2.61, df = 12, *p* = 0.11). Seroprevalence was significantly higher in the red knot *Calidris canutus* (77.5, 95% CI: 70.2–83.4; Z value = 3.93, p<0.001) as compared to the ruddy turnstone (47.1, 95% CI: 36.8–57.5) and significantly lower in the dunlin (1.4, 95% CI: 0.6–3.3; Z value = −7.17, p<0.001) and the sanderling *Calidris alba* (4.8, 95% CI: 0.2–22.7; Z value = −2.53, p<0.05) sampled concurrently at the Banc d'Arguin (Figure 3, supporting information Table S5). No AIV antibodies were found in species sampled in the Inner Niger Delta, in either Eurasian migrants (ruff *Philomachus pugnax*, wood sandpiper *Tringa glareola*) or Afrotropical species (African jacana, painted snipe *Rostratula benghalensis,* spur-winged lapwing *Vanellus spinosus*) (in supporting information Table S4). As in the analysis of AIV infection rate we found that the sampling occasion accounted for most of the variance of the random part (variance estimation = 0.41) while the inclusion of each of the other random factors (site and year) did not improve the model fit (LRT, p>0.5).

The limited number of species in the data set precluded tests of the relationship between species traits and variation in seroprevalence in our modelling analysis. We note, however, that a high seroprevalence was found in both one mainly tactile (red knot) and one mainly visual-foraging species (ruddy turnstone) at the Banc d'Arguin and that globally, no difference was found between species using these two distinct foraging strategies (χ2 = 0.45, df = 1, *p* = 0.50). Among species originating from Arctic breeding ground and wintering in the coastal environment of the Banc d'Arguin we found both species with high (red knot, ruddy turnstone) and low seroprevalence (dunlin, sanderling).

## Discussion

Despite an unprecedentedly large geographic and taxonomic coverage we did not detect any hotspots of AIV infection in shorebirds that matched the criterion that infection rate should be consistently elevated and about an order of magnitude greater than in other sites (≥10%). We did find a relatively high infection rate at one of our sampling sites - Lakes Manyame-Chivero, Zimbabwe - on three distinct occasions, representing different seasons (Sep. and Nov.- late dry season, Jan.- wet season) and years (2007 and 2008). These two adjacent lakes, though of relatively small area (65 and 185 km2, respectively) represent a key site for shorebirds in Zimbabwe [23] and AIV has been detected continuously in the wild bird community present at these lakes [33]. The relatively low number of individuals sampled per species and sampling occasions limit our ability to identify the species associated with this potential hotspot. The African jacana was the shorebird species that was most commonly sampled and the most frequently found infected at the Lakes Manyame-Chivero, but surprisingly no African jacana was found infected at any other sites across Africa despite a relatively large number of individuals sampled (n = 312 birds). Follow-up sampling studies conducted at Lakes Manyame-Chivero in following years during the same season and on the same species consistently detected AIV-positive birds but at a lower infection rate, making us unable to confirm the existence of a recurrent AIV hotspot at this site. Peaks in AIV prevalence may be associated with very narrow seasonal windows (e.g., few weeks in May at the Delaware Bay [6], [9]). However the timing of these seasonal windows may be more variable in tropical than in temperate ecosystems. The high variability of seasonal rainfall in the tropics and the related fluctuations in the timing of reproduction and congregation of waterbirds may produce different seasonal dynamics of AIV infection between years. The inter-annual difference in prevalence measured at Lakes Manyame-Chivero may result from a difference in lake level and the related difference in the local density of waterbirds [33].

The overall absence of shorebird-AIV hotspots in our study is notable for several reasons. First, we looked for hotspots in a remarkably large number of species (n = 69) from ten shorebird families, including species and families that had never been tested for AIV infection before. This wide exploration identified many new AIV host species. Second, the majority of birds were tested for AIV infection from both cloacal and oropharyngeal samples. As predicted, a significantly higher prevalence was found in birds tested using both types of sample than those using one type of sample only. Among birds (n = 4711) tested for both cloacal and oropharyngeal samples, few birds were found positive concurrently for both types of sample (n = 3), while birds were found positive as frequently from cloacal samples only (n = 34) as from oropharyngeal samples only (n = 36) (McNemar's chi-squared = 0.01, df = 1, p-value = 0.90). Moreover, the two viruses isolated in this study originated from both an oropharyngeal (dunlin/H1N1) and a cloacal sample (spotted redshank/H10N7). Though these results confirm the importance of the respiratory tract for the replication of AIV in shorebirds as previously shown for wild ducks [2], [19], [20], neither the inclusion of new species nor the collection and testing of oropharyngeal swabs revealed new AIV hotspots. Major attention was given to the preservation of the cold chain from the field to the laboratory, although in some instances logistical constrains in remote field locations or unexpected international shipment delays may account for the low virus isolation rate obtained. However detection of AIV by rRT-PCR is insensitive to differences in cold-chain conditions and freeze-thaw cycles [34] therefore differences in storage conditions should not have affected our results.

**Figure 4:**
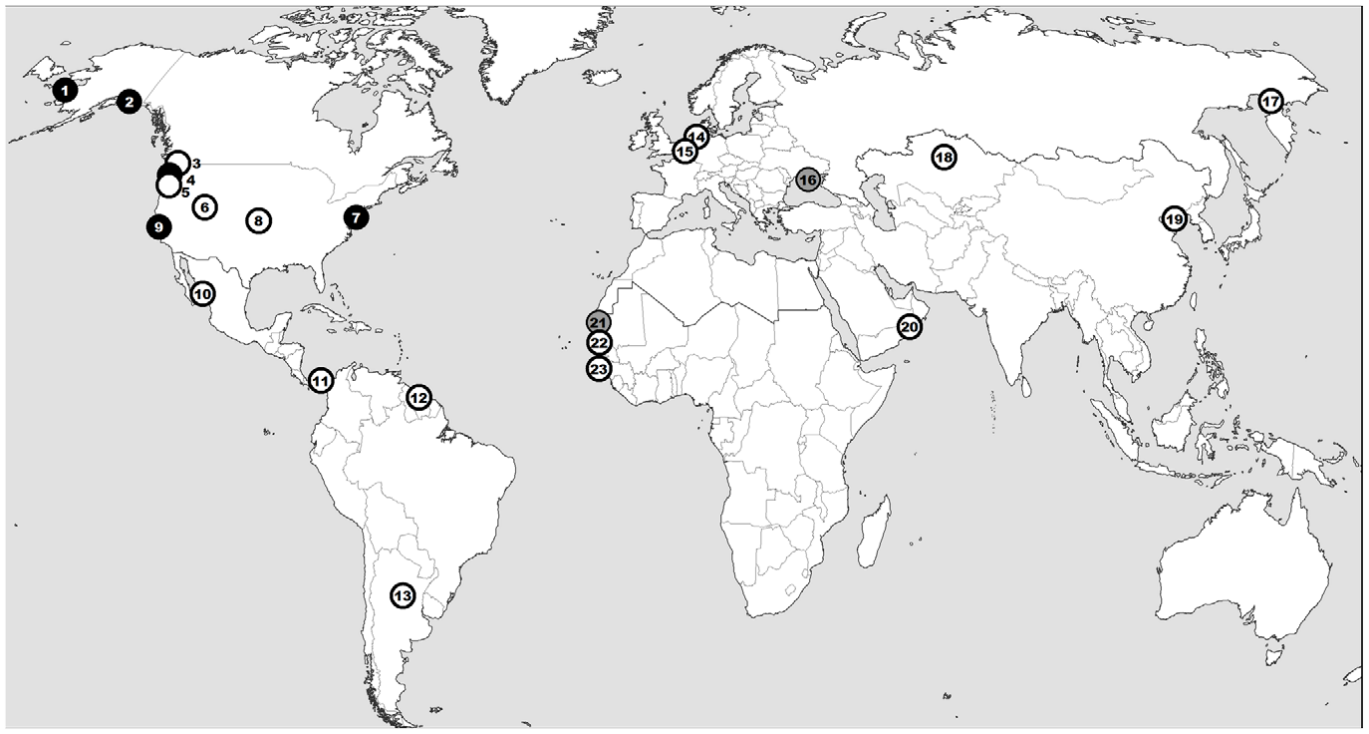
Location of the world's largest congregation sites of waders (sandpipers, plovers and allies), i.e. sites where at least 500,000 birds congregate annually. Among these sites where birds have been tested for AIV infection (black symbols - from the literature; grey symbols - this study) an AIV hotspot has been reported only at the Delaware Bay (no. 7). See Table S6 (supporting information) for detailed information on each site. 1-Yukon-Kuskokwim Delta, 2-Copper River Delta, 3-Fraser' River Estuary, 4-Gray's harbour estuary, 5-Bay of Fundy, 6-Great Salt Lake, 7-Delaware Bay, 8-Cheyenne Bottoms, 9-San Francisco Bay, 10-Bahia de Santa Maria, 11-Upper Bay of Panama, 12-Suriname coast, 13-Laguna Mar Chiquita, 14-Wadden Sea, 15-Rhine-Maas-Schelde Delta, 16-Azov Sea, 17-Sea of Okhotsk, 18-Tengiz-Korgalzhyn Lakes, 19-Yellow Sea coast, 20-Arabian Sea off Oman, 21-Banc d'Arguin, 22-Senegal River Delta, 23-Bijagos Archipelago (map by M. Gély ©Cirad).

Third, we found a consistently low AIV prevalence in shorebirds in several sites that are, like Delaware Bay, large seasonal congregation sites of shorebirds. Prevalence was also not related to the abundance classes of shorebirds across all our sampling sites. With two million wintering shorebirds, about one-third of the entire East Atlantic Flyway population, the Banc d'Arguin clearly qualifies as one of the larger congregation sites of shorebirds in the world. Shorebirds forage on the intertidal flats of the Banc d'Arguin at a density four times as high as the average density recorded in the other major wintering sites along the East-Atlantic coast [26]. Despite those ecological characteristics, we found a consistently low AIV prevalence in shorebirds, including in ruddy turnstone, sampled in various seasons and years at the Banc d'Arguin. We found a similarly low prevalence at other major shorebird congregation sites, including the Sivash (Crimea peninsula, Ukraine) and the Senegal River delta (Senegal-Mauritania). In Figure 4 we present the distribution of the world's largest congregation sites of waders (i.e. shorebirds of the Scolpaci (sandpipers and allies) and Charadrii (plovers and allies) clades): AIV detection studies in these birds have been conducted for seven of these sites (including two from this study) but a high infection rate has been reported only at Delaware Bay (in supporting information Table S6). Therefore, a large congregation of birds appears to be an insufficient condition for the existence of a hotspot of AIV infection.

Another characteristic of Delaware Bay is the exceptionally high number of ruddy turnstones which represent the second most abundant shorebird species at this site (annual maximum counts range from c. 32,000 to 105,000 birds [35]). No other site worldwide supports such a concentration of ruddy turnstones. By comparison, the highest abundance of this species over Western Eurasia and Africa is found at the Banc d'Arguin in winter, with maximum annual counts fluctuating between c. 4,100 and 10,300 individuals [23]. During spring migration, shorebirds gather at Delaware Bay to feed on the eggs of horseshoe crabs (*Limulus polyphemus*) spawning in the bay. Variation in spawning activity and wave action creates patches of egg concentration where shorebirds forage in densities as high as 210 birds/m^2^ [36]. Much lower shorebird densities are found at other sites where resources are more uniformly distributed over the intertidal zone (e.g. 7×10^−3^ birds/m^2^ on average at the Banc d'Arguin [26]). The exceptionally high abundance of ruddy turnstones, the unusually high foraging density of shorebirds and the potential variation in their feeding strategy associated with a unique food source (horseshoe crab eggs) may explain the specific AIV hotspot at the Delaware Bay.

Our repeated sampling revealed a low but continuous circulation of AIVs in shorebirds at the Banc d'Arguin throughout the wintering period of Eurasian migrants. This coastal tropical site combines several constraints to virus persistence in the environment: high temperatures, solar radiations and wind exposure, salinity, little precipitations, and tidal washing of the tidal flats. The main maintenance hosts of AIVs (the dabbling ducks of the *Anas* genus) are also absent. The perpetuation of AIV throughout the wintering period, therefore, likely results from a continuous inter-individual transmission among shorebirds facilitated by the aggregation of birds into large flocks at a few high tide roosts. Besides its importance as a wintering site, the Banc d'Arguin is also a major staging site for migratory shorebirds: it represents the tip of a flyway funnel draining migratory birds breeding across the extensive Arctic tundra of North America and Eurasia through the Atlantic coast from Western Europe down to southern Africa (Figure 1). Continuous AIV circulation at such a migration crossroad creates the potential for reassortment between AIV strains originating from different geographic areas as well as the potential for geographically extensive dispersal of new viruses. Globally, our results reveal a low but widespread circulation of AIV in shorebirds across Eurasian and Afro-tropical regions. AIV-infected shorebirds were detected in a large number of species, in all study regions and in both inland-freshwater and coastal-saline environments. This finding contrast with the absence of AIVs previously reported in studies of shorebirds in northern Europe [3], [4] and suggest that these birds play a role, as in the Americas, in the epidemiology of AIV in the old word.

Results from our study also reveal that the ruddy turnstone is not consistently associated with AIV hotspots. Prevalence measured in this species was low and not significantly different from the prevalence measured in cohabiting species. Our multivariate analyses revealed the existence of significant species variations in AIV prevalence and seroprevalence. Contrary to our predictions, prevalence and seroprevalence were no lower in visual-foraging species (feeding mostly by pecking in small foraging flocks) than in tactile-foraging species (mainly probing in large foraging flocks). Nor were they lower in Arctic-coastal species that remain year-round in AIV-poor environments than in boreal-freshwater species (see methods). Conversely, no AIV antibodies were found in freshwater Eurasian or African shorebird species. This absence is striking, given that most of these freshwater shorebirds cohabit, sometimes in close proximity and high numbers, with potentially infectious wild ducks in boreal, temperate, and/or Afro-tropical wetlands.

AIV-specific antibodies acquired after a natural infection have been reported to persist generally for not more than a year in captive mallards [37] and in free-ranging migratory geese [38]. Similarly in this study we found no difference in seroprevalence between first-year and adults birds. Though the potential existence of long-lasting antibodies in shorebirds cannot be completely ruled out, the very high AIV antibody prevalence but low infection rate that we found at the Banc d'Arguin in red knot (c.80%) and ruddy turnstone (c. 50%) suggest that these species experienced a prior exposure to a relatively high AIV infection rate at other staging sites along their annual range. A similar pattern of high antibody prevalence (c. 50–90%, [8], [31]) and low infection rate (<1%, [5], [9]) was found in red knot at Delaware Bay, also suggesting a prior high AIV infection rate at other sites. These three bird populations have non-overlapping breeding ranges: the sub-species of red knot that winter or stop at the Banc d'Arguin (*canutus*) breed on the Taimyr Peninsula, western Siberia, whereas the sub-species that stop at Delaware Bay (*rufa*) breed in the central Canadian Arctic [30]; the ruddy turnstone of the Banc d'Arguin breed in both the north-eastern Canada and the Fennoscandia-west Russia regions [23]. Very little information is available on the infection status of these populations along their migratory flyway, and hence the site and season where potentially high infection rates occur – i.e. potential AIV hotspots - remain to be discovered.

Highly contrasting seroprevalence values were found at the Banc d'Arguin among four species - red knot, ruddy turnstone, dunlin and sanderling - that are ecologically and phylogenetically highly related. A very similar pattern in seroprevalence variation was found among the same species sampled at Delaware Bay [8], [9], [31] (Figure 3). The two species found with a high seroprevalence at both sites - red knot, ruddy turnstone – both breed in the high-Arctic, while closely related species with a lower seroprevalence have a more southerly breeding distribution (except the sanderling, Figure 3). This finding is in contradiction with the adjustment to disease pressure hypothesis that predicts a lower exposure to pathogens and a lower investment in immune system in species breeding at higher latitudes as an adaptation to pathogen-poor environment and a trade-off to compensate the higher energetic costs associated with long-distance migration and breeding in climatically adverse conditions [30], [39]. Studies conducted in the high Arctic regions (Svalbard [38], Chukchi Peninsula [40], Northern Alaska [41], Taimyr Peninsula [42]) have consistently reported no or very low AIV infection rate in wild birds. The low seroprevalence found in the sanderling – one of the more northerly breeding shorebird species – in both the Banc d'Arguin and Delaware Bay (Figure 3) also suggests that the high Arctic is not the region where high AIV infection occurs.

Differences in seroprevalence among these closely related shorebird species do not appear to be readily explained by environmental variables. They may instead result from an intrinsic difference between species in their receptivity to AIV infection, and/or in their ability to mount and maintain an acquired antibody-mediated (humoral) immune response. At Delaware Bay the low infection rate in several shorebird species commingling with ruddy turnstone at very high density to forage on the same egg resource also suggest the existence of species-level constraints to interspecies AIV transmission. The spectrum and the distribution of sialic acid receptors of AIV on host epithelial tissues varies substantially among closely related bird species [22], [43] and may lead to variations in permissiveness for infection and limit transmission between cohabiting species. Species-specific differences in acquired immune responses have also been found among closely related shorebird species after an experimental infection with the same antigens, with ruddy turnstone showing higher antibody responses than sanderling, ruff and red knot [44]. The lower immune response of red knot compared to ruddy turnstone does not, however, fit with the high AIV antibody prevalence found in these two species.

On few occasions the serological status of birds individually identified from their ring number could be controlled on consecutive sampling occasions at the Banc d'Arguin NP. One dunlin seroconverted between November 2009 and March 2010, as a result of an infection that probably occurred at the Banc d'Arguin, since this site constitutes the larger southernmost staging site of dunlin of its migration flyway [23]. AIV was also detected by rRT-PCR in a red knot sampled in March 2010 that had been previously found seropositive in November 2009; this bird was still seropositive in March 2010. This suggests that acquired immunity is only partial and that a prior exposure does not fully protect against a subsequent AIV infection.

In summary, our study reveals that, when considered separately, the individual features associated with a disease hotspot do not systematically produce a locally and temporally high transmission rate in other contexts. Outside Delaware Bay, the ruddy turnstone has not been found infected at a higher prevalence than other shorebird species (in supporting information Table S1). In addition, no AIV-hotspot has been found at any of the other world's largest shorebird congregation sites investigated so far (Figure 4, supporting information Table S6). Different constituents should be combined to generate an exceptionally high transmission rate. To what extent the constituents (species, environment, and season) of AIV hotspots are identical and temporally stable, hence predictable, remains to be elucidated [1]. More generally, we suggest that interpreting existing hotspots in light of data from other ecosystems and pathogens should help to understand and work towards a more general model of hotspots.

## Supporting Information

Figure S1 **Number of AIV-positive birds detected per species for a given sampling occasion compared to the threshold number of positive birds (solid line) below which the prevalence is unlikely (probability <0.05) to be greater than 10% for a sample of the same size.** Points on or above the line represent potential species-hotspots, i.e. species for a given sampling occasion (n = 11) for which the number of positive birds was too large for rejecting the hypothesis that prevalence could be >10%. Only species sampling occasions (n = 89) that had at least 28 birds sampled were considered in this analysis.
(DOCX)

Table S1 **Summary of the worldwide investigation of AIV infection in ruddy turnstone.**
(DOCX)

Table S2 **List of sampling sites ranked by latitude and sampling details.**
(DOCX)

Table S3 **Overview of the shorebird species tested for AIV infection († species reported infected with AIV for the first time).**
(DOCX)

Table S4 **Seroprevalence of AIV antibodies in shorebird species sampled at the Banc d'Arguin (Mauritania) and the Inner Niger Delta (Mali).**
(DOCX)

Table S5 **Results** **of the model selection procedure relating variations in seroprevalence of AIV antibodies to species ecological traits in shorebirds sampled at two West African sites (Banc d'Arguin, Mauritania; Inner Niger Delta, Mali).**
(DOCX)

Table S6 **List of the world's largest wader congregation sites ranked by latitude on all continents.** These sites correspond to locations that support at least 500,000 waders annually selected from Internationally Important Sites databases (main sources: America - Western Hemisphere Shorebird Network; Africa and Western Eurasia - Delany et al., 2009; Australasia - Bamford et al., 2008). Sites where AIV infection studies in waders have been conducted are presented in bold with details about AIV detection. Waders include all species from the Scolopaci (sandpipers, snipes, phalaropes, jacanas) and Charadrii clades (plovers, oystercatchers, stilts).
(DOCX)
