# Supplementary material for: Investigating Avian Influenza Infection Hotspots in Old-World Shorebirds
Source: PLoS One. 2012 Sep 28;7(9):e46049. doi: 10.1371/journal.pone.0046049 (PMC3460932; doi:10.1371/journal.pone.0046049)
Supplement: Figure S1 — Number of AIV-positive birds detected per species for a given sampling occasion compared to the threshold number of positive birds (solid line) below which the prevalence is unlikely (probability <0.05) to be greater than 10% for a sample of the same size. Points on or above the line represent potential species-hotspots, i.e. species for a given sampling occasion (n = 11) for which the number of positive birds was too large for rejecting the hypothesis that prevalence could be >10%. Only species sampling occasions (n = 89) that had at least 28 birds sampled were considered in this analysis. (DOCX) [file pone.0046049.s001.docx]

Figure S1. Number of AIV-positive birds detected per species for a given sampling occasion compared to the threshold number of positive birds (solid line) below which the prevalence is unlikely (probability <0.05) to be greater than 10% for a sample of the same size. Points on or above the line represent potential species-hotspots, i.e. species for a given sampling occasion (n= 11) for which the number of positive birds was too large for rejecting the hypothesis that prevalence could be > 10%. Only species sampling occasions (n= 89) that had at least 28 birds sampled were considered in this analysis.
