# Supplementary material for: Investigating Avian Influenza Infection Hotspots in Old-World Shorebirds
Source: PLoS One. 2012 Sep 28;7(9):e46049. doi: 10.1371/journal.pone.0046049 (PMC3460932; doi:10.1371/journal.pone.0046049)
Supplement: Table S1 — Summary of the worldwide investigation of AIV infection in ruddy turnstone. (DOCX) [file pone.0046049.s002.docx]

Table S1. Summary of the worldwide investigation of AIV infection in ruddy turnstone.

| Study area | Year | Season | Diagn. method | Sample type | No. birds | No. pos. | Ref ^c^ |
| --- | --- | --- | --- | --- | --- | --- | --- |
| USA, Delaware Bay | 1985-87 | Year round | Virus isolation | Fecal or cloacal | 162 | 38 (23.5%) | [1] |
| USA, Delaware Bay | 2000-05 | May-Jun. | Virus isolation | Cloacal | 4533 | 540 (11.9%) | [2,3] |
| Mauritania, Banc d’Arguin | 2006-2010 | Nov.-Apr. | rRT-PCR | Oral-pharyn. + cloacal | 158 | 1(0.6%) | This study |
| USA, Alaska | 2006-10 | Jun.-Sep. | rRT-PCR | Oral-pharyn. + cloacal | 142 | 1(0.7%) | [4] |
| Peru, Pacific coast | 2006-07 | Year round | Virus isolation | Fecal | 81 | 2(2.5%) | [5] |
| European Union ^a^ | 2006 | Year round | rRT-PCR | Cloacal | 51 | 0 | [6] |
| Northern Europe, North America | 1998-2006 | - | rRT-PCR | Cloacal | 26 | 0 | [7] |
| Bermuda | 2002 | Dec. | Virus isolation | Cloacal | 16 | 0 | [2] |
| USA, Florida | 2001 | May | Virus isolation | Cloacal | 3 | 0 | [2] |
| USA, Georgia | 2000 | Sep. | Virus isolation | Cloacal | 2 | 0 | [2] |
| Germany | 2001-02 | Jul.-Oct. | Virus isolation | Oral-pharyn. + cloacal | 1 | 0 | [8] |
| Russia, Chukchi Penins. (Siberia) | 2005 | Aug. | rRT-PCR | Cloacal | 1 | 0 | [9] |
| USA, Pacific coast | 2006 | Jul.-Dec. | rRT-PCR | Cloacal | 1 | 0 | [10] |
| USA, Pacific coast | 2006-07 | Year round | rRT-PCR | Cloacal | 1 | 0 | [11] |
| Mongolia, Western region | 2003-2008 | Aug.-Sep. | Virus isolation | Cloacal | 1 | 0 | [12] |
| Total - Delaware Bay ^b^ |  |  |  |  | 4695 | 578 (12.3%) |  |
| Total - Outside Delaware Bay ^b^ |  |  |  |  | 484 | 4 (0.8%) |  |

a. Six countries: Belgium (n=2), Germany (n=3), Greece (n=3), Malta (n=2), Netherlands (n=36), Sweden (n=8).

b. Additional samples had been collected from various shorebird species including ruddy turnstone at Delaware Bay [13,14] and in Eastern Germany [15] but detailed information on infection rate at the species level is not available.

c. List of references:

1. Kawaoka Y, Chambers TM, Sladen WL, Webster RG (1988) Is the gene pool of influenza viruses in shorebirds and gulls different from that in wild ducks? Virology 163: 247–250.
2. Hanson BA, Luttrell MP, Goekjian VH, Niles L, Swayne DE, et al. (2008) Is the occurrence of avian influenza virus in Charadriiformes species and location dependent? J Wildl Dis 44: 351–361.
3. Stallknecht DE, Luttrell MP, Poulson R, Goekjian V, Niles L, et al. (2012) Detection of avian influenza viruses from shorebirds: evaluation of surveillance and testing approaches. J Wildl Dis 48: 382-393.
4. USFWS/USGS (2007-2011) Sampling for highly pathogenic Asian H5N1 avian influenza in migratory birds in Alaska: results of 2006 (2007, 2008, 2009 and 2010) field season. Progress Report, U.S. Fish and Wildlife Service (Region 7, Alaska) U.S. Geological Survey, Alaska Science Center, Anchorage, Alaska, and U.S. Geological Survey, National Wildlife Health Center, Madison, Wisconsin.
5. Ghersi BM, Blazes DL, Icochea E, Gonzalez RI, Kochel T, et al. (2009) Avian influenza in wild birds, central coast of Peru. Emerg Infect Dis 15: 935–938.
6. European Commission (2006) Annual report of the European Union avian influenza surveillance in wild bird, 2006. Annex VII. Prepared by the Community Reference Laboratory for avian influenza and Newcastle disease. SANCO/10194/2007 REV 1.
7. Munster VJ, Baas C, Lexmond P, Waldenstrom J, Wallensten A, et al. (2007) Spatial, temporal, and species variation in prevalence of influenza A viruses in wild migratory birds. PLoS Pathog 3: e61.
8. Hlinak A, Mühle RU, Werner O, Globig, A, Starick E, et al. (2006) A virological survey in migrating waders and other waterfowl in one of the most important resting sites of Germany. J Vet Med B 53: 105–110.
9. Wahlgren J, Waldenstrom J, Sahlin S, Haemig PD, Fouchier RAM, et al. (2008) Gene segment reassortment between American and Asian lineages of avian influenza virus from waterfowl in the Beringia area. Vector Borne Zoonotic Dis 8: 783–790.
10. Iverson SA, Takekawa JY, Schwarzbach S, Cardona CJ, Warnock N, et al. (2008) Low prevalence of avian influenza virus in shorebirds on the Pacific Coast of North America. Waterbirds 31:602-610
11. Dusek RJ, Bortner JB, De Liberto TJ, Hoskins J, Franson JC, et al. (2009) Surveillance for high pathogenicity avian influenza virus in wild birds in the Pacific Flyway of the United States, 2006–2007. Avian Dis 53:222–30.
12. Marchenko VY, Alekseev AY, Tserennorov D, Yurlov AK, Susloparov IM, et al. (2010) Results of the Influenza Virus Surveillance in Wild Birds in Western Part of Mongolia. Asian Pacific Journal of Tropical Medicine 3: 90-93.
13. Krauss S, Walker D, Pryor SP, Niles L, Chenghong L, et al. (2004) Influenza A viruses of migrating wild aquatic birds in North America. Vector Borne Zoonotic Dis 4: 177–189.
14. Krauss S, Stallknecht DE, Negovetich NJ, Niles LJ, Webby RJ, et al. (2010) Coincident ruddy turnstone migration and horseshoe crab spawning creates an ecological 'hot spot' for influenza viruses. Proc R Soc B 277: 3373-3379.
15. Süss J, Schafer J, Sinnecker H, Webster RG (1994) Influenza virus subtypes in aquatic birds of eastern Germany. Arch Virol 135: 101–114.
