# Supplementary material for: Investigating Avian Influenza Infection Hotspots in Old-World Shorebirds
Source: PLoS One. 2012 Sep 28;7(9):e46049. doi: 10.1371/journal.pone.0046049 (PMC3460932; doi:10.1371/journal.pone.0046049)
Supplement: Table S2 — List of sampling sites ranked by latitude and sampling details. (DOCX) [file pone.0046049.s003.docx]

Table S2. List of sampling sites ranked by latitude and sampling details.

| Country | Site | Lat. | Long. | Environment | Abundance^a^ | No. sampling occasions | Year | Months of sampling | | | | | | | | | | | | No. Species | No. Birds | Lab^b^ |
| --- | --- | --- | --- | --- | --- | --- | --- | --- | --- | --- | --- | --- | --- | --- | --- | --- | --- | --- | --- | --- | --- | --- |
|  |  |  |  |  |  |  |  | J | F | M | A | M | J | J | A | S | O | N | D |  |  |  |
| Ukraine | Eastern Sivash | 45.7 | 34.4 | Saline | 4 | 2 | 2006 |  |  |  | X |  |  |  |  |  | X |  |  | 14 | 685 | CIRAD, CRL |
| Romania | Danube delta | 45.0 | 29.2 | Freshwater | 3 | 1 | 2006 |  |  |  |  |  |  |  |  |  |  | X |  | 5 | 11 | CIRAD |
| Turkey | Kızılırmak delta | 41.6 | 36.1 | Saline | 2 | 1 | 2006 |  |  |  |  |  |  |  |  |  |  | X |  | 12 | 145 | CIRAD |
|  | Yumurtalık Lagoons | 36.7 | 35.7 | Saline | 2 | 1 | 2006 |  |  |  |  |  |  |  |  |  |  | X |  | 8 | 147 | CIRAD |
| Iran | Fereydoon Kenar marshes | 36.7 | 52.5 | Freshwater | 2 | 1 | 2007 |  | X |  |  |  |  |  |  |  |  |  |  | 3 | 6 | CIRAD |
| Morocco | Marais du Bas Loukkos | 35.2 | -6.1 | Freshwater | 1 | 1 | 2006 |  | X |  |  |  |  |  |  |  |  |  |  | 2 | 30 | IZS |
|  | Sidi Moussa-Oualidia Lagoon | 32.8 | -8.9 | Saline | 2 | 1 | 2007 |  |  |  |  |  |  |  |  |  |  |  | X | 17 | 234 | CIRAD |
| Tunisia | Thyna salt pans | 34.6 | 10.7 | Saline | 2 | 2 | 2006-07 |  |  |  | X | X |  |  |  |  |  |  |  | 9 | 92 | CIRAD, SEPRL |
| Egypt | Nile river delta | 31.3 | 32.0 | Saline | 3 | 3 | 2006-08 | X |  | X | X | X |  |  |  |  |  |  |  | 16 | 458 | CIRAD, FLI, NAMRU |
|  | Lake Qarun | 29.5 | 30.6 | Saline | 2 | 1 | 2008 |  |  |  | X |  |  |  |  |  |  |  |  | 1 | 28 | FLI |
| Mauritania | Banc d’Arguin National Park | 20.1 | -16.3 | Saline | 4 | 5 | 2006-10 |  | X | X | X |  |  |  |  |  |  | X | X | 18 | 2018 | CIRAD, IZS |
| Senegal- Mauritania | Senegal river delta | 16.4 | -16.4 | Saline | 4 | 2 | 2006, 2010 |  | X | X |  |  |  |  |  |  |  |  |  | 1 | 192 | CIRAD, IZS |
| Sudan | El Saggay Island (Nile river) | 16.0 | 32.6 | Freshwater | 2 | 1 | 2007 |  |  | X |  |  |  |  |  |  |  |  |  | 5 | 204 | CIRAD |
| Mali | Inner Niger Delta | 15.3 | -4.3 | Freshwater | 3 | 7 | 2006-09 | X | X |  |  |  | X |  |  | X | X |  |  | 26 | 1001 | CIRAD, IZS |
| Niger | Kurfunkura pond (Maradi) | 13.3 | 7.2 | Freshwater | 1 | 1 | 2006 |  |  | X |  |  |  |  |  |  |  |  |  | 2 | 8 | IZS |
|  | Gaya (Niger river) | 12.1 | 3.2 | Freshwater | 1 | 1 | 2006 |  |  | X |  |  |  |  |  |  |  |  |  | 2 | 4 | IZS |
| Chad | Lake Chad | 12.9 | 14.9 | Freshwater | 3 | 1 | 2006 |  | X |  |  |  |  |  |  |  |  |  |  | 4 | 10 | IZS |
| Nigeria | Hadejia-Nguru wetlands | 12.8 | 10.8 | Freshwater | 3 | 1 | 2007 |  | X |  |  |  |  |  |  |  |  |  |  | 8 | 83 | CIRAD |
| Burkina | Lake Kompienga | 11.2 | 0.6 | Freshwater | 1 | 1 | 2006 |  | X |  |  |  |  |  |  |  |  |  |  | 13 | 151 | IZS |
| Ethiopia | Lake Debre Zeit | 8.7 | 38.8 | Freshwater | 1 | 2 | 2007-08 | X |  |  |  |  |  |  |  |  |  |  |  | 14 | 187 | CIRAD,IZS |
| South Sudan | Bargel wetland | 7.1 | 29.4 | Freshwater | 2 | 1 | 2008 |  |  |  |  |  |  |  |  |  |  |  | X | 2 | 13 | FLI |
| Kenya | Lakes around Nairobi | -1.2 | 37.0 | Freshwater | 2 | 1 | 2006 |  | X |  |  |  |  |  |  |  |  |  |  | 7 | 59 | OVI |
| Tanzania | Lake Manyara | -3.7 | 35.8 | Saline | 3 | 1 | 2007 |  | X |  |  |  |  |  |  |  |  |  |  | 5 | 333 | FLI |
| Malawi | Lake Chilwa | -15.2 | 35.5 | Freshwater | 2 | 2 | 2006-07 |  | X |  |  |  |  |  |  |  |  |  | X | 6 | 31 | CIRAD, OVI |
| Zambia | Kafue Flats | -15.8 | 27.3 | Freshwater | 3 | 1 | 2006 |  |  |  |  |  |  |  |  |  |  | X |  | 11 | 149 | CIRAD |
| Zimbabwe | Lakes Manyame-Chivero | -17.8 | 30.6 | Freshwater | 1 | 15 | 2007-10 | X |  | X |  | X |  | X | X | X |  | X |  | 21 | 755 | OVI |
| Botswana | Lake Ngami | -20.4 | 22.8 | Freshwater | 2 | 4 | 2007-08 |  | X |  |  |  | X |  |  |  | X | X |  | 17 | 276 | OVI |
| Mozambique | Massingir Dam | -23.9 | 31.9 | Freshwater | 1 | 1 | 2007 |  |  |  |  |  |  |  | X |  |  |  |  | 4 | 13 | OVI |
|  | Lake Chuali | -25.0 | 32.9 | Freshwater | 1 | 4 | 2007-08 |  |  | X |  | X |  | X |  |  |  | X |  | 12 | 177 | OVI |
| South Africa | Barberspan wetland | -26.6 | 25.6 | Freshwater | 2 | 12 | 2007-09 |  | X |  | X |  | X | X | X |  | X | X | X | 19 | 144 | OVI |
|  | Strandfontein | -34.1 | 18.5 | Freshwater | 2 | 11 | 2007-09 | X |  | X |  | X |  | X |  | X |  | X | X | 8 | 71 | OVI |
| Total | 31 |  |  |  |  | 89 | 2006-10 |  |  |  |  |  |  |  |  |  |  |  |  | 65 | 7715 | 7 |

a. Maximum total number of birds of all shorebird species; four abundance classes: (1) <5,000; (2) 5,000-50,000; (3) 50,000-500,000; (4) >500,000 birds.

b. CIRAD (Centre de Coopération Internationale en Recherche Agronomique pour le Développement, Montpellier, France), CRL (Community Reference Laboratory, VLA, Weybridge, UK), FLI (Friedrich-Loeffler-Institut, Greifswald-Insel Riems, Germany), IZS (Istituto Zooprofilattico Sperimentale delle Venezie, Padova, Italia); NAMRU (US Naval Medical Research Unit-3, Cairo, Egypt), OVI (Onderstepoort Veterinary Institute, Agricultural Research Council, RSA; SEPRL (Southeast Poultry Research Laboratory, USDA/ARS, USA).
