# Supplementary material for: Investigating Avian Influenza Infection Hotspots in Old-World Shorebirds
Source: PLoS One. 2012 Sep 28;7(9):e46049. doi: 10.1371/journal.pone.0046049 (PMC3460932; doi:10.1371/journal.pone.0046049)
Supplement: Table S3 — Overview of the shorebird species tested for AIV infection († species reported infected with AIV for the first time). (DOCX) [file pone.0046049.s004.docx]

Table S3. Overview of the shorebird species tested for AIV infection († species reported infected with AIV for the first time).

| Family |  |  | Origin | No. birds (No. birds pos.) | | | | % pos. (total) |
| --- | --- | --- | --- | --- | --- | --- | --- | --- |
|  | Species |  |  | Double | Cloacal | Oropharyn. | Total |  |
| Scolopacidae | *24 species* |  |  | 2654 (33) | 917 (7) | 643 (5) | 4214 (45) | 1.1 |
|  | Dunlin | *Calidris alpina* | Eurasian | 772 (9) | 255 | 445 (3) | 1472 (12) | 0.8 |
|  | Little stint | *Calidris minuta†* | Eurasian | 573 (8) | 80 | 11 | 664 (8) | 1.2 |
|  | Ruff | *Philomachus pugnax* | Eurasian | 303 (3) | 284 (3) | 5 | 592 (6) | 1.0 |
|  | Red knot | *Calidris canutus* | Eurasian | 197 (4) | 26 | 127 (2) | 350 (6) | 1.7 |
|  | Wood sandpiper | *Tringa glareola†* | Eurasian | 185 (1) | 80 | 9 | 274 (1) | 0.4 |
|  | Common redshank | *Tringa totanus* | Eurasian | 203 | 12 | 2 | 217 | 0.0 |
|  | Ruddy turnstone | *Arenaria interpres* | Eurasian | 143 (1) | 8 | 9 | 160 (1) | 0.6 |
|  | Curlew sandpiper | *Calidris ferruginea†* | Eurasian | 28 | 48 (2) | 12 | 88 (2) | 2.3 |
|  | Greenshank | *Tringa nebularia* | Eurasian | 37 | 18 |  | 55 | 0.0 |
|  | Common snipe | *Gallinago gallinago* | Eurasian | 21 | 31 |  | 52 | 0.0 |
|  | Sanderling | *Calidris alba* | Eurasian | 37 | 4 | 8 | 49 | 0.0 |
|  | Common sandpiper | *Actitis hypoleucos†* | Eurasian | 30 (1) | 15 | 3 | 48 (1) | 2.1 |
|  | African snipe | *Gallinago nigripennis†* | Afrotropical | 31 (2) | 1 | 3 | 35 (2) | 5.7 |
|  | Marsh sandpiper | *Tringa stagnatilis* | Eurasian | 16 | 15 | 1 | 32 | 0.0 |
|  | Bar-tailed godwit | *Limosa lapponica* | Eurasian | 22 | 1 | 7 | 30 | 0.0 |
|  | Spotted redshank | *Tringa erythropus†* | Eurasian | 7 (1) | 23 (2) |  | 30 (3) | 10.0 |
|  | Green sandpiper | *Tringa ochropus†* | Eurasian | 18 (1) |  |  | 18 (1) | 5.6 |
|  | Temminck’s stint | *Calidris temmincki* | Eurasian | 13 | 4 |  | 17 | 0.0 |
|  | Jack snipe | *Lymnocryptes minimus†* | Eurasian | 4 (1) | 6 | 1 | 11 (1) | 9.1 |
|  | Black-tailed godwit | *Limosa limosa* | Eurasian | 6 (1) | 4 |  | 10 (1) | 10.0 |
|  | Great snipe | *Gallinago media* | Eurasian | 4 |  |  | 4 | 0.0 |
|  | Eurasian curlew | *Numenius arquata* | Eurasian | 3 |  |  | 3 | 0.0 |
|  | Broad-billed sandpiper | *Limicola falcinellus* | Eurasian |  | 2 |  | 2 | 0.0 |
|  | Lesser yellowlegs | *Tringa flavipes* | Eurasian | 1 |  |  | 1 | 0.0 |
| Laridae | *16 species* |  |  | 271 (1) | 1130 (20) | 22 | 1423 (21) | 1.5 |
|  | Slender-billed gull | *Chroicocephalus genei* | Euras.-Afro. | 199 (1) | 287 (13) | 22 | 508 (14) | 2.8 |
|  | Black-backed gull | *Larus fuscus†* | Eurasian | 1 | 247 (1) |  | 248 (1) | 0.4 |
|  | Terns | *Sterna sp* | Euras.-Afro. |  | 330 (3) |  | 330 (3) | 0.9 |
|  | Mediterranean gull | *Larus melanocephalus* | Eurasian |  | 131 |  | 131 | 0.0 |
|  | Gull-billed tern | *Gelochelidon nilotica* | Euras.-Afro. |  | 70 |  | 70 | 0.0 |
|  | White-winged tern | *Chlidonias leucopterus* | Eurasian |  | 51 (3) |  | 51 (3) | 5.9 |
|  | King gull | *Larus hartlaubii* | Afrotropical | 29 |  |  | 29 | 0.0 |
|  | Grey-headed gull | *Chroicocephalus cirrocephalus* | Afrotropical | 12 | 8 |  | 20 | 0.0 |
|  | Kelp gull | *Larus dominicanus* | Afrotropical | 11 |  |  | 11 | 0.0 |
|  | Black-headed gull | *Chroicocephalus ridibundus* | Eurasian | 11 |  |  | 11 | 0.0 |
|  | Whiskered tern | *Chlidonias hybridus* | Eurasian | 1 | 4 |  | 5 | 0.0 |
|  | Little tern | *Sterna albifrons* | Euras.-Afro. | 2 | 2 |  | 4 | 0.0 |
|  | Common tern | *Sterna hirundo* | Eurasian | 3 |  |  | 3 | 0.0 |
|  | Yellow-legged gull | *Larus michaellis* | Eurasian | 1 |  |  | 1 | 0.0 |
|  | Sandwich tern | *Sterna sandvicensis* | Eurasian | 1 |  |  | 1 | 0.0 |
| Charadriidae | *15 species* |  |  | 808 (23) | 97 | 28 (1) | 933 (24) | 2.6 |
|  | Blacksmith lapwing | *Vanellus armatus†* | Afrotropical | 261 (12) | 18 |  | 288 (12) | 4.2 |
|  | Kittlitz’s plover | *Charadrius pecuarius* | Afrotropical | 176 | 38 | 9 | 221 | 0.0 |
|  | Spur-winged lapwing | *Vanellus spinosus* | Afrotropical | 126 (1) | 16 |  | 142 (1) | 0.7 |
|  | Kentish plover | *Charadrius alexandrinus†* | Eurasian | 48 (3) | 2 | 4 (1) | 50 (3) | 6.0 |
|  | Common ringed plover | *Charadrius hiaticula†* | Eurasian | 42 | 4 | 1 | 50 (1) | 2.0 |
|  | Grey plover | *Pluvialis squatarola†* | Eurasian | 36 (1) | 8 |  | 48 (1) | 2.1 |
|  | Three-banded plover | *Charadrius tricollaris†* | Afrotropical | 37 (2) | 1 | 3 | 39 (2) | 5.1 |
|  | Little ringed plover | *Charadrius dubius†* | Eurasian | 23 (1) | 1 | 7 | 24 (1) | 4.2 |
|  | Chestnut-banded plover | *Charadrius pallidus* | Afrotropical | 18 |  | 4 | 18 | 0.0 |
|  | Wattled lapwing | *Vanellus senegallus†* | Afrotropical | 14 (3) |  |  | 17 (3) | 17.6 |
|  | Eurasian golden plover | *Pluvialis apricaria* | Eurasian | 11 |  |  | 11 | 0.0 |
|  | Black-headed lapwing | *Vanellus tectus* | Afrotropical | 8 | 1 |  | 9 | 0.0 |
|  | Long-toed lapwing | *Vanellus crassirostris* | Afrotropical | 1 | 7 |  | 8 | 0.0 |
|  | Northern lapwing | *Vanellus vanellus* | Eurasian | 5 | 1 |  | 6 | 0.0 |
|  | Crowned lapwing | *Vanellus coronatus* | Afrotropical | 2 |  |  | 2 | 0.0 |
| Jacanidae | *2 species* |  |  | 615 (15) | 52 | 34 (1) | 701 (16) | 2.3 |
|  | African jacana | *Actophilornis africana†* | Afrotropical | 608 (15) | 52 | 34 (1) | 694 (16) | 2.3 |
|  | Lesser jacana | *Microparra capensis* | Afrotropical | 7 |  |  | 7 | 0.0 |
| Glareolidae | *2 species* |  |  | 182 (1) | 9 | 3 | 194 (1) | 0.5 |
|  | Collared pratincole | *Glareola pratincola†* | Euras.-Afro. | 182 | 6 | 3 | 191 (1) | 0.5 |
|  | Temminck’s courser | *Cursorius temminckii* | Afrotropical | (1) | 3 |  | 3 | 0.0 |
| Rostratulidae | Greater painted-snipe | *Rostratula benghalensis* | Afrotropical | 136 | 17 | 1 | 154 | 0.0 |
| Recurvirostridae | *2 species* |  |  | 33 | 50 |  | 154 | 0.0 |
|  | Black-winged stilt | *H. himantopus* | Euras.-Afro. | 23 | 50 |  | 83 | 0.0 |
|  | Pied avocet | *Recurvirostra avosetta* | Euras.-Afro. | 10 |  |  | 73 | 0.0 |
| Haematopidae | *2 species* |  |  | 6 |  |  | 10 | 0.0 |
|  | African oystercatcher | *Haematopus moquini* | Afrotropical | 4 |  |  | 6 | 0.0 |
|  | Eurasian oystercatcher | *Haematopus ostralegus* | Eurasian | 2 |  |  | 4 | 0.0 |
| Rynchopidae | African skimmer | *Rynchops flavirostris* | Afrotropical | 4 |  |  | 2 | 0.0 |
| Burhinidae | Water thick-knee | *Burhinus vermiculatus* | Afrotropical | 2 |  | 1 | 4 | 0.0 |
| Total |  | *All species* |  | 4711 (73) | 2272 (27) | 732 (7) | 4 | 1.4 |
