# Supplementary material for: Investigating Avian Influenza Infection Hotspots in Old-World Shorebirds
Source: PLoS One. 2012 Sep 28;7(9):e46049. doi: 10.1371/journal.pone.0046049 (PMC3460932; doi:10.1371/journal.pone.0046049)
Supplement: Table S4 — Seroprevalence of AIV antibodies in shorebird species sampled at the Banc d'Arguin (Mauritania) and the Inner Niger Delta (Mali). (DOCX) [file pone.0046049.s005.docx]

Table S4. Seroprevalence of AIV antibodies in shorebird species sampled at the Banc d’Arguin (Mauritania) and the Inner Niger Delta (Mali)

| Site | Species |  | Sampling date | No. birds (No. birds pos.) | | % pos. (total) |
| --- | --- | --- | --- | --- | --- | --- |
|  |  |  |  | per occasion | total |  |
| Banc d’Arguin | Dunlin | *Calidris alpina* | Apr. 2008 | 71 (1) | 352 (5) | 1.4 |
|  |  |  | Nov. 2009 | 176 (3) |  |  |
|  |  |  | Mar. 2010 | 105 (1) |  |  |
|  | Red knot | *Calidris canutus* | Nov. 2009 | 15 (5) | 151 (117) | 77.5 |
|  |  |  | Mar. 2010 | 136 (112) |  |  |
|  | Ruddy turnstone | *Arenaria interpres* | Apr. 2008 | 52 (27) | 85 (40) | 47.1 |
|  |  |  | Nov. 2009 | 5 |  |  |
|  |  |  | Mar. 2010 | 28 (13) |  |  |
|  | Sanderling | *Calidris alba* | Nov. 2009 | 5 | 21 (1) | 4.8 |
|  |  |  | Mar. 2010 | 16 (1) |  |  |
|  | Slender-billed gull | *Chroicocephalus* *genei* | Apr. 2008 | 1 | 6 (1) | 16.7 |
|  |  |  | Mar. 2010 | 5 (1) |  |  |
|  | Curlew sandpiper | *Calidris ferruginea* | Nov. 2009 | 1 | 2 | 0.0 |
|  |  |  | Mar. 2010 | 1 |  |  |
|  | Common ringed plover | *Charadrius hiaticula* | Nov. 2009 | 2 | 2 | 0.0 |
|  | Lesser black-backed gull | *Larus fuscus* | Nov. 2009 | 1 | 1 | 0.0 |
|  | Bar-tailed godwit | *Limosa lapponica* | Nov. 2009 | 1 | 1 | 0.0 |
| Inner Niger Delta | Ruff | *Philomachus pugnax* | Feb. 2009 | 85 | 89 | 0.0 |
|  |  |  | Oct. 2009 | 4 |  |  |
|  | Wood sandpiper | *Tringa glareola* | Feb. 2009 | 24 | 82 | 0.0 |
|  |  |  | Oct. 2009 | 58 |  |  |
|  | African jacana | *Actophilornis africana* | Feb. 2009 | 29 | 48 | 0.0 |
|  |  |  | Jun. 2009 | 19 |  |  |
|  | Greater painted-snipe | *Rostratula benghalensis* | Feb. 2009 | 8 | 24 | 0.0 |
|  |  |  | Jun. 2009 | 11 |  |  |
|  |  |  | Oct. 2009 | 5 |  |  |
|  | Spur-winged lapwing | *Vanellus spinosus* | Feb. 2009 | 6 | 20 | 0.0 |
|  |  |  | Oct. 2009 | 14 |  |  |
|  | Little ringed plover | *Charadrius dubius* | Feb. 2009 | 7 | 13 | 0.0 |
|  |  |  | Oct. 2009 | 6 |  |  |
|  | Black-headed lapwing | *Vanellus tectus* | Oct. 2009 | 9 | 9 | 0.0 |
|  | Collared pratincole | *Glareola pratincola* | Feb. 2009 | 8 | 8 | 0.0 |
|  | Kittlitz’s plover | *Charadrius pecuarius* | Feb. 2009 | 3 | 7 | 0.0 |
|  |  |  | Jun. 2009 | 4 |  |  |
|  | Common ringed plover | *Charadrius hiaticula* | Feb. 2009 | 2 | 2 | 0.0 |
|  | Common snipe | *Gallinago gallinago* | Feb. 2009 | 1 | 2 | 0.0 |
|  |  |  | Oct. 2009 | 1 |  |  |
|  | Great snipe | *Gallinago media* | Feb. 2009 | 2 | 2 | 0.0 |
|  | Little stint | *Calidris minuta* | Oct. 2009 | 1 | 1 | 0.0 |
|  | Common sandpiper | *Actitis hypoleucos* | Oct. 2009 | 1 | 1 | 0.0 |
|  | Black-winged stilt | *Himantopus himantopus* | Feb. 2009 | 1 | 1 | 0.0 |
| Total |  |  |  |  | 930 (164) | 17.6 |
