# Supplementary material for: Investigating Avian Influenza Infection Hotspots in Old-World Shorebirds
Source: PLoS One. 2012 Sep 28;7(9):e46049. doi: 10.1371/journal.pone.0046049 (PMC3460932; doi:10.1371/journal.pone.0046049)
Supplement: Table S5 — Results of the model selection procedure relating variations in seroprevalence of AIV antibodies to species ecological traits in shorebirds sampled at two West African sites (Banc d'Arguin, Mauritania; Inner Niger Delta, Mali). (DOCX) [file pone.0046049.s006.docx]

Table S5. Results of the model selection procedure relating variations in seroprevalence of AIV antibodies to species ecological traits in shorebirds sampled at two West African sites (Banc d’Arguin, Mauritania; Inner Niger Delta, Mali).

| Explanatory variables | Coefficient ± S.E. | χ2 | df | p |
| --- | --- | --- | --- | --- |
| Age |  | 0.79 | 2 | 0.67 |
| Species |  | 281.31 | 8 | < 0.001 |
| Sanderling *C. alba* | *-2.75* ± 1.09* |  |  |  |
| Dunlin *C. alpina* | -3.74 ± 0.52*** |  |  |  |
| Red knot *C. canutus* | 1.48 ± 0.38*** |  |  |  |

All models were fitted as generalized mixed effects models, with sampling occasion fitted as random intercept terms to control for pseudo-replication and other explanatory variables as fixed effect. The initial full model was simplified by backwards elimination of non-significant variables starting with variable that had the lowest explanatory power (highest P values). The test statistics refer to a log-likelihood ratio test between the model in which the variable is retained and in which it is excluded. Coefficient estimates are given only for individual species for which seroprevalence estimates was statistically different from Ruddy Turnstones (t-test, ***p<0.001,*p<0·05).
