# Supplementary material for: Investigating Avian Influenza Infection Hotspots in Old-World Shorebirds
Source: PLoS One. 2012 Sep 28;7(9):e46049. doi: 10.1371/journal.pone.0046049 (PMC3460932; doi:10.1371/journal.pone.0046049)
Supplement: Table S6 — List of the world's largest wader congregation sites ranked by latitude on all continents. These sites correspond to locations that support at least 500,000 waders annually selected from Internationally Important Sites databases (main sources: America - Western Hemisphere Shorebird Network; Africa and Western Eurasia - Delany et al., 2009; Australasia - Bamford et al., 2008). Sites where AIV infection studies in waders have been conducted are presented in bold with details about AIV detection. Waders include all species from the Scolopaci (sandpipers, snipes, phalaropes, jacanas) and Charadrii clades (plovers, oystercatchers, stilts). (DOCX) [file pone.0046049.s007.docx]

Table S6. List of the world’s largest wader congregation sites ranked by latitude on all continents. These sites correspond to locations that support at least 500,000 waders annually selected from Internationally Important Sites databases (main sources: America - Western Hemisphere Shorebird Network; Africa and Western Eurasia - Delany et al., 2009; Australasia - Bamford et al., 2008). Sites where AIV infection studies in waders have been conducted are presented in bold with details about AIV detection. Waders include all species from the Scolopaci (sandpipers, snipes, phalaropes, jacanas) and Charadrii clades (plovers, oystercatchers, stilts).

| N° | Wader congregation sites | | | | | AIV infection | | | | |
| --- | --- | --- | --- | --- | --- | --- | --- | --- | --- | --- |
|  | Location | Country (state/province) | Lat. / Long. | Most abundant species ^a^ | Ref.^b^ | No. birds  sampled | No. pos. (%) | Main species ^a^  (*AIV-positive species) | Sampling date | Ref.^c^ |
| **North America** | |  |  |  |  |  |  |  |  |  |
| **1** | **Yukon-Kuskokwim**  **Delta** | **USA (Alaska)** | 62.6° N 164.9° W | Bar-tailed godwit | [1] | 3821 | 3 (0.1) | Dunlin, Rock and Sharp-tailed sandpipers, Bar-tailed godwit* |  | [11] |
| **2** | **Copper River Delta** | **USA (Alaska)** | 60.5° N 145.0° W | Western sandpiper, Dunlin | [1,2] | 146 | 1 (0.7) | Western sandpiper* | Spring 2006-07 | [12] |
|  |  |  |  |  |  | 1050 | 0 | Western and Least sandpipers | Spring 2006-07 | [13] |
| 3 | Fraser' River Estuary | Canada (British Columbia) | 49.1° N 123.2° W | Western sandpiper | [1] |  |  |  |  |  |
| **4** | **Gray's harbour estuary** | **USA (Washington)** | 46.9° N  124.0° W | Western and Least sandpipers, Dunlin, Semipalmated and Black-bellied plovers, Red knot | [1] | 907 | 2 (0.2) | Western sandpiper*, Dunlin* | Spring 2006-07, Autumn 2006-07 | [12] |
| 5 | Bay of Fundy | Canada (New Brunswick/Nova Scotia) | 45.1˚ N  64.3˚ W | Semipalmated sandpiper | [1] |  |  |  |  |  |
| 6 | Great Salt Lake | USA (Utah) | 40°-42° N 112-113° W | Wilson’s phalarope, American avocet, Black-necked stilt | [1] |  |  |  |  |  |
| **7** | **Delaware Bay** | **USA (New Jersey/Delaware)** | 39.1° N 75.3° W | Semipalmated sandpiper, Ruddy turnstone, Red knot, Sanderling | [1,3] | 1446 ^d^ | 96 (6.6) ^d^ | Ruddy turnstone*, Red knot*, Semipalmated sandpiper*, Sanderling* ^d^ | Spring 1985-87, Autumn 1985-86 | [14] |
|  |  |  |  |  |  | 133 | 1 (0.8) | Red knot* | Spring 2005 | [15] |
|  |  |  |  |  |  | 7343 ^d^ | 463 (6.3)^d^ | Red knot, Ruddy turnstone, Sanderlings, Semipalmated sandpiper | Spring 1988-2008, Autumn 1985-1988 | [16] |
|  |  |  |  |  |  | 10892 ^d^ | 611 (5.6)^d^ | Ruddy turnstone*, Red knot*, Sanderling*, Semipalmated sandpiper*, Dunlin*, short-billed dowitcher* | Spring 2000-09 | [17,18] |
| 8 | Cheyenne Bottoms | USA (Kansas) | 38.5° N 98.6° W | White-rumped, Baird’s and Stilt sandpiper, Long-billed dowitchers and Wilson’s phalaropes | [1] |  |  |  |  |  |
| **9** | **San Francisco Bay** | **USA (California)** | 37.4° N 122.0° W | Black-bellied and Semipalmated plovers, Willet, Long-billed curlew, Marbled godwit | [1] | 792 | 6 (0.8) | Western sandpiper*, Long-billed dowitcher* | Spring 2006-07, Autumn 2006 | [12] |
| 10 | Bahia de Santa Maria | Mexico (Sinaloa) | 24.4°-25.5° N, 107.5°-108.4° W | Western sandpiper, American avocet | [1,4] |  |  |  |  |  |
| **South America** | |  |  |  |  |  |  |  |  |  |
| 11 | Upper Bay of Panama | Panama | 9.0˚ N  79.0˚ W | Semipalmated and Western sandpipers, Semipalmated plover, Short-billed dowitcher, Willet | [1] |  |  |  |  |  |
| 12 | Suriname coast (Bigi Pan, Coppename Monding and Wia Wia NR) | Suriname | 5.9˚ N, 54.9° -56.7˚ W | Semipalmated sandpiper, Semipalmated plover, Short-billed dowitcher, Greater and Lesser Yellowlegs | [1] |  |  |  |  |  |
| 13 | Laguna Mar Chiquita | Argentina (Cordoba) | 30.7° S  62.5° W | Wilson's phalarope, Lesser yellowlegs, White-rumped sandpiper | [1] |  |  |  |  |  |
| **Europe** | |  |  |  |  |  |  |  |  |  |
| 14 | Wadden Sea | The Netherlands, Germany, Denmark | 53°- 55° N 5°- 9° W | Red knot, Dunlin, Bar-tailed godwit | [5] |  |  |  |  |  |
| 15 | Rhine-Maas-Schelde Delta | The Netherlands | 51.3° N, 4.0° E | Eurasian oystercatcher, Northern lapwing, Eurasian golden plover, Eurasian curlew | [5] |  |  |  |  |  |
| **16** | **Azov Sea**  **(The Sivash)** | **Ukraine (Crimea)** | 46.1°N, 34.3°E | Ruff, Dunlin, Pied avocet, Curlew sandpiper | [5] | 685 | 1 (0.1) | Dunlin*, Ruff | Spring 2006, Autumn 2006 | this study |
| **Asia** | |  |  |  |  |  |  |  |  |  |
| 17 | Sea of Okhotsk (Moroshechnaya and Penzhina Rivers Estuary) | Russia (Kamchatka) | 57.0° - 62.5° N 156.0 -164.5° E | Dunlin, Red-necked stint, Red-necked phalarope, Great knot, Bar-tailed godwit | [6,7] |  |  |  |  |  |
| 18 | Tengiz-Korgalzhyn Lakes | Kazakhstan | 50.3° N, 69.3°E | Red-necked phalarope, Ruff, Little stint, Dunlin | [5] |  |  |  |  |  |
| 19 | Yellow Sea coast (Yancheng, Haung He and Yalu Jiang NNR, Mangyeung and Dongjin estuaries) | North and South Korea, China | 31.0° - 41.0° N  11.7°- 12.6° E | Dunlin, Great knot, Bar-tailed godwit, Black-bellied plover | [7-10] |  |  |  |  |  |
| 20 | Arabian Sea off Oman | Oman | 16.5° - 22°N, 53- 59.5°E | Lesser sandplover, Eurasian curlew, Bar-tailed godwit, Dunlin | [5] |  |  |  |  |  |
| **Africa** | |  |  |  |  |  |  |  |  |  |
| **21** | **Banc d’Arguin** | **Mauritania** | 19.5°-20°N, 16°-16.5°W | Dunlin, Bar-tailed godwit, Red knot, Curlew sandpiper | [5] | 1379 | 12 (0.9) | Dunlin*, Red knot*, Ruddy turnstone* | Spring 2006,08,10  Autumn 2006,09 | this study |
| 22 | Senegal River Delta | Mauritania, Senegal | 16.3°N, 16.1°E | Ruff, Black-winged stilt, Black-tailed godwit | [5] | *192 ^e^* | *13 (6.8) ^e^* | *Slender-billed gull* ^e^* | *Spring 2006, 10* | *this study* |
| 23 | Bijagos Archipelago | Guinea-Bissau | 11.2° N, 16.0° E | Bar-tailed godwit, Red Knot, Curlew sandpiper, Little stint | [5] |  |  |  |  |  |

1. Scientific names: Eurasian oystercatcher *Haematopus ostralegus*, American avocet *Recurvirostra americana*, Pied avocet *Recurvirostra avosetta*, Black-necked stilt *Himantopus himantopus*, Lesser sandplover *Charadrius mongolus*, Semipalmated plover *Charadrius semipalmatus*, Northern lapwing *Vanellus vanellus*, Eurasian golden plover *Pluvialis apricaria*, Black-bellied plover *Pluvialis squatarola*, Rock sandpiper *Calidris ptilocnemis*, White-rumped sandpiper *Calidris fuscicollis*, Baird’s sandpiper *Calidris bairdii*, Stilt sandpiper *Calidris himantopus*, Sanderling *Calidris alba*, Ruff *Philomachus pugnax*, Curlew sandpiper *Calidris ferruginea*, Semipalmated sandpiper *Calidris pusilla*, Western sandpiper *Calidris mauri*, Least sandpipers *Calidris minutilla*, Dunlin *Calidris alpina*, Little stint *Calidris minuta*, Sharp-tailed sandpiper *Calidris acuminata*, Red-necked stint *Calidris ruficollis*, Great knot *Calidris tenuirostris*, Red knot *Calidris canutus*, Long-billed dowitcher *Limnodromus scolopaceus*, Short-billed dowitcher *Limnodromus griseus*, Bar-tailed godwit *Limosa lapponica*, Black-tailed godwit *Limosa limosa*, Long-billed curlew *Numenius americanus*, Eurasian curlew *Numenius numenius*, Marbled godwit *Limosa fedoa*, Willet *Catoptrophorus semipalmatus*, Greater yellowlegs *Tringa melanoleuca*, Lesser Yellowlegs *Tringa flavipes*, Ruddy turnstone *Arenaria interpres*, Red-necked phalarope *Phalaropus lobatus*, Wilson’s phalarope *Phalaropus tricolor*.
2. References of shorebird abundance:
3. Western Hemisphere Shorebird Network (WHSRN). List of sites. Available: http://www.whsrn.org/sites/list-sites
4. Bishop MA, Meyers PM, McNeley PF (2000) A method to estimate migrant shorebird numbers on the Copper River Delta, Alaska. J Field Ornithol 71: 627–637.
5. Clark K E, Niles LJ, Burger J (1993) Abundance and distribution of migrant shorebirds in Delaware Bay. Condor 95: 694–705
6. Egilis A, Oring LW, , Carrera E, Nelson JW, Martinez Lopez A (1998) Shorebirds surveys in Ensenada pabellones and Bahia Santa Maria, Sinaloa, Mexico : critical winter habitats for pacific flyway shorebirds. Wilson Bull 110: 332-341.
7. Delany S, Scott D, Dodman T, Stroud D (2009) An Atlas of Wader Populations in Africa and Western Eurasia. Wageningen: Wetlands International. 524p
8. Gerasimov YN, Huettmann F (2006) Shorebirds of the Sea of Okhotsk: status and overview. Stilt 50:15–22.
9. Bamford M, Watkins D, Bancroft W, Tischler G, Wahl J (2008) Migratory Shorebirds of the East Asian - Australasian Flyway; Population Estimates and Internationally Important Sites. Canberra: Wetlands Inter­national.
10. Barter MA (2002) Shorebirds of the Yellow Sea: Importance, threats and conservation status. Global Series 9, International Wader Studies 12. Canberra: Wetlands International.
11. Delany S, Reyes C, Hubert E, Pihl S, Rees E, et al. (1999) Results from the International Waterbird Census in the Western Palearctic and Southwest Asia 1995 and 1996. Wageningen: Wetlands International.
12. Wilson JR, Barter MA (1998) Identification of potentially important staging areas for ‘‘long jump’’ migrant waders in the East-Australasian Flyway during northward migration. Stilt 32:16-27.
13. References of AIV infection studies:
14. USFWS/USGS (2007-2011) Sampling for highly pathogenic Asian H5N1 avian influenza in migratory birds in Alaska: results of 2006 (2007, 2008, 2009 and 2010) field season. Progress Report, U.S. Fish and Wildlife Service (Region 7, Alaska) U.S. Geological Survey, Alaska Science Center, Anchorage, Alaska, and U.S. Geological Survey, National Wildlife Health Center, Madison, Wisconsin.
15. Iverson SA, Takekawa JY, Schwarzbach S, Cardona CJ, Warnock N, et al. (2008) Low prevalence of avian influenza virus in shorebirds on the Pacific Coast of North America. Waterbirds 31:602-610
16. Winker K, Spackman E, Swayne DE (2008) Rarity of influenza A virus in spring shorebirds, southern Alaska. Emerg Infect Dis 14: 1314–1316.
17. Kawaoka Y, Chambers TM, Sladen WL, Webster RG (1988) Is the gene pool of influenza viruses in shorebirds and gulls different from that in wild ducks? Virology 163: 247–250.
18. Munster VJ, Baas C, Lexmond P, Waldenstrom J, Wallensten A, et al. (2007) Spatial, temporal, and species variation in prevalence of influenza A viruses in wild migratory birds. PLoS Pathog 3: e61.
19. Krauss S, Stallknecht DE, Negovetich NJ, Niles LJ, Webby RJ, et al. (2010) Coincident ruddy turnstone migration and horseshoe crab spawning creates an ecological 'hot spot' for influenza viruses. Proc R Soc B 277: 3373-3379.
20. Hanson BA, Luttrell MP, Goekjian VH, Niles L, Swayne DE, et al. (2008) Is the occurrence of avian influenza virus in Charadriiformes species and location dependent? J Wildl Dis 44: 351–361.
21. Stallknecht DE, Luttrell MP, Poulson R, Goekjian V, Niles L, et al. (2012) Detection of avian influenza virsues from shorebirds: evaluation of surveillance and testing approaches. J Wildl Dis 48: 382-393.
22. Include some gull and tern species (Laughing gull* *Larus atricilla*, Herring gull* *L. argentatus*, Ring-billed gull *L. delawarensis*, Great black-backed gull *L. marinus*, Royal tern *Sterna maxima* and Common tern *S. hirundo*).
23. Only Laridae were tested at this site (Slender-billed gull *Chroicocephalus genei*)
